# Cellular and Extracellular MicroRNA Dysregulation in LRRK2-Linked Parkinson’s Disease

**DOI:** 10.1007/s12035-025-05379-2

**Published:** 2025-11-27

**Authors:** Felix Knab, Jun-Hoe Lee, Raja Nirujogi, Kevin Menden, Luca Braunger, Lambrianna Logarnudi, Benjamin Riebenbauer, Fatma Busra Isik, Anto Praveen Rajkumar, Stefan Czemmel, Julia Fitzgerald, Thomas Gasser, Christian Johannes Gloeckner

**Affiliations:** 1 Department of Neurodegeneration, Hertie Institute for Clinical Brain Research, University of Tuebingen, Tuebingen, Germany; 2 Quantitative Biology Center (QBiC), University of Tuebingen, Tuebingen, Germany; 3 MRC Protein Phosphorylation and Ubiquitylation Unit, University of Dundee, Dundee, Scotland; 4 German Center for Neurodegenerative Diseases (DZNE), Tuebingen, Germany; 5 Institute of Mental Health, Mental Health and Clinical Neurosciences Academic Unit, University of Nottingham, Nottingham, UK

**Keywords:** Parkinson’s disease, Biomarker, Micro-RNA, LRRK2, IPSCs

## Abstract

Cell-free microRNAs in body fluids have emerged as promising biomarker candidates in neurodegenerative diseases. While several studies have identified dysregulated miRNAs in sporadic Parkinson’s disease, it remains unclear whether distinguishable alterations of cell-free miRNAs occur in genetic forms of the disease, such as those associated with the LRRK2 G2019S mutation. In this proof-of-concept study, we used a human induced pluripotent stem cell-derived dopaminergic neuron model to investigate whether the LRRK2 G2019S mutation induces detectable changes in the intra- and extracellular miRNAome, and whether miRNA signatures identified in vitro can be validated in patient-derived cerebrospinal fluid. We differentiated dopaminergic neurons from induced pluripotent stem cells carrying the LRRK2 G2019S mutation and an isogenic gene-corrected control. Extracellular vesicles were isolated from the culture medium and used as a source of cell-free miRNA. Next, small RNA libraries were generated and analyzed. Differentially expressed microRNAs were validated in an independent batch using RT-qPCR. We further quantified candidate microRNAs in cerebrospinal fluid samples from five LRRK2 G2019S patients and matching healthy controls. The patient cohort included the fibroblast donor from whom the stem cells were originally derived. We successfully isolated extracellular vesicles from induced pluripotent stem cell-derived human dopaminergic neurons. We identified a distinct set of differentially expressed miRNAs in cellular and cell-free RNA, among which let-7g-5p and miR-21-5p were consistently upregulated and validated across independent replicates. These alterations were reflected in the cerebrospinal fluid of the original donor and partially reproduced in additional LRRK2 patients, supporting the concept of patient-specific signatures. A strong correlation between intra- and extracellular miRNA expression was observed. Our findings demonstrate that induced pluripotent stem cell-derived dopaminergic neurons can serve as a model to identify individualized, cell-free microRNA signatures associated with the LRRK2 G2019S mutation. The dysregulated miRNAs detected in vitro were mirrored in patient cerebrospinal fluid, supporting their potential as accessible molecular readouts. These results lay the groundwork for personalized biomarker strategies in genetic forms of Parkinson’s disease and warrant further validation in larger patient cohorts.

**Supplementary Information:**

The online version contains supplementary material available at 10.1007/s12035-025-05379-2.

## Introduction

Parkinson’s disease (PD) is a neurodegenerative disorder with an estimated total of over six million affected patients worldwide [1]. Diagnosing PD, particularly in its early stages, remains a clinical challenge [2]. There is a great need for non-invasive markers identified in body fluids, as they hold promise for enabling cost-effective, widely accessible tools to detect clinical onset or monitor disease progression. While PD was long considered sporadic, 10–15% of cases follow Mendelian inheritance, with over ten genes linked to familial PD (fPD) [3]. Mutations in the Leucine-rich repeat kinase 2 (LRRK2) gene are the most common genetic cause of fPD and are also found in ~ 2% of sporadic cases due to reduced penetrance [4–6]. This suggests that LRRK2 plays an important role in the pathogenesis of at least a specific subset of both sporadic PD (sPD) and fPD patients. Its pathogenic relevance is often linked to altered kinase activity, commonly assessed via the phosphorylated Rab10 (pRab10) to total Rab10 (tRab10) ratio [7, 8]. With LRRK2 inhibitors rapidly approaching clinical testing, a LRRK2-related non-invasive readout of target engagement or treatment efficacy would be of immense interest.

MicroRNAs (miRNAs) are small non-coding RNAs that regulate gene expression post-transcriptionally through mRNA degradation or translational repression [9]. Numerous miRNAs are dysregulated in neurodegenerative diseases, including PD and Alzheimer’s disease (AD) [10]. While each has a distinct pathology, several miRNAs are consistently altered across disorders. In PD, many dysregulated miRNAs are implicated in α-synuclein regulation, neuronal survival, and inflammation. For instance, several miRNAs target the SNCA gene (encoding α-synuclein), reducing α-synuclein protein levels by inhibiting translation or promoting mRNA decay [11]. Compared to other molecular biomarkers such as messenger RNAs, cell-free DNA (cfDNA), epigenetic markers, and proteins, miRNAs offer several key advantages. They are remarkably stable in extracellular environments, largely due to their protection by carriers such as extracellular vesicles (EVs) or Argonaute proteins, which shield them from RNase-mediated degradation [12, 13]. cfDNA is predominantly released during cell death and thus reflects ongoing neurodegeneration only once substantial neuronal loss has already occurred [14]. In contrast, miRNA dysregulation has been observed even in early or prodromal stages of neurodegenerative diseases [15]. Their dynamic nature allows them to respond to changing pathophysiological states in real time, offering insights into processes such as inflammation, synaptic dysfunction, and protein homeostasis [16]. In contrast to relatively stable epigenetic changes that require complex analysis, miRNA profiling is technically accessible and reproducible. Quantification is typically achieved via standardized qPCR platforms, with commercially available primers covering most known miRNAs. This facilitates high-throughput analysis and enables the development of multi-marker panels. Protein-based assays, by comparison, often depend on the availability and specificity of antibodies, which may be lacking for novel or low-abundance targets.

While various mechanisms of active secretion into the extracellular space are being investigated [17–19], it appears most of the cell-free RNA is bound to protein complexes such as Argonaute. A small proportion of cell-free RNA estimated at around 3% is vesicle-associated [20]. EVs are membrane-enclosed vesicles with a size of 40 to 1000 nm that are secreted by cells and contain cytosolic components such as proteins and nucleic acids [21]. EVs have been extensively investigated in biomarker research due to their accessibility from biofluids [22], and numerous studies have reported differential expression of EV-associated miRNAs in biofluids from PD patients. For instance, early work on CSF-derived EVs identified miRNAs that differentiated PD patients from controls with high accuracy [23]. More recently, Li et al. applied next-generation sequencing and machine learning to miRNA expression levels quantified in plasma EVs from PD, idiopathic REM sleep behavior disorder, and controls, revealing distinct miRNA signatures for each group and underscoring the diagnostic potential of EV-miRNA profiles [24].

A large number of miRNAs have been computationally predicted to target LRRK2 [25] and mutant LRRK2 is known to disrupt the cellular miRNA landscape [26, 27]. However, only a limited subset of these interactions has been experimentally confirmed and the overall number of studies focusing on LRRK2-associated changes in miRNAs remains small. Tolosa et al. used a predefined array to quantify miRNAs in human dopaminergic neurons (hDaNs) from sPD and LRRK2 G2019S patients and healthy controls. A set of miRNAs differed between PD and controls, but no differences were found between sPD and fPD [28]. Botta et al. applied the same array to serum samples from PD patients with and without the G2019S mutation. Dysregulated miRNAs were validated in two independent PD cohorts, with no significant differences between sporadic and familial cases, similar to the findings of Tolosa et al. [29]. A third study profiled serum miRNAs in symptomatic and asymptomatic G2019S carriers, sporadic PD patients, and controls, distinguishing between symptomatic and asymptomatic mutation carriers [30]. We recently published a study identifying distinct miRNA signatures in serum that differentiate LRRK2 mutation carriers from sPD patients and healthy controls [31]. Despite these studies, to date, the miRNA content of neuronal extracellular vesicles in the context of LRRK2-linked PD remains entirely unexplored.

This study aimed to characterize the miRNA signature of neuronal EVs in the context of a LRRK2 G2019S mutation and to explore individualized target identification by detecting candidate miRNAs in a patient-derived cell line and validating them in the CSF of the same individual. We differentiated hDaNs from a previously characterized induced pluripotent stem cell (iPSC) line derived from a LRRK2 G2019S patient [32, 33] and generated small RNA libraries from neuronal EVs. miRNA profiles were compared to those of an isogenic gene-corrected control. To assess concordance with intracellular miRNA levels, we also profiled miRNAs in cellular RNA from the same hDaNs. Finally, we analyzed CSF from a small cohort of LRRK2 G2019S PD patients, including the donor of the original iPSC line.

The present study differs from previous work in several key aspects. It is the only one using an isogenic control line, ensuring that observed differences are due solely to the LRRK2 mutation and not genetic background—an important advantage in small-scale cell models. Unlike prior studies relying on predefined arrays, we employed an unbiased small RNA library approach, allowing the discovery of novel miRNA targets. Most notably, we focused on neuronal EVs as a source of miRNAs. While cellular miRNA profiles provide mechanistic insights, they do not reveal whether such changes are detectable in patients. Neuronal EVs, by contrast, can be isolated from CSF, offering direct CNS proximity, and even from serum, enabling less invasive analysis [34]. Moreover, our approach is unique in its translational design: candidate miRNAs identified in a patient-derived cell line were validated in the CSF of the same individual, bridging in vitro discovery with patient-level confirmation.

## Materials and Methods

### Differentiation of Human Dopaminergic Neurons from Neuronal Progenitor Cells

Two lines of neural progenitor cells (NPCs) carrying the LRRK2 G2019S mutation or the gene-corrected LRRK2 wild type were previously generated from patient-derived iPSCs [32, 33]. Here, L1 and L2 refer to the two PD patients carrying the LRRK2 G2019S mutation from whom cells were derived. L1 Mut and L2 Mut denote the cell lines carrying the LRRK2 G2019S mutation, while L1 GC and L2 GC represent the gene-corrected isogenic control lines. When referring to both lines derived from a single patient (e.g., L1 GC and L1 Mut), we use the terms L1 lines or L2 lines, respectively. Initially, we had used both of these lines for the generation of the small RNA libraries and for further experiments. Upon closer characterization of both lines, due to drastically decreased protein levels of LRRK2 in the L2 Mut line, we decided to focus on results from the L1 lines throughout this study. Results from the L2 lines will be reported in the supplemental Materials and Methods.

NPCs were cultured in base media (50% neurobasal (Thermo Fisher Scientific (Waltham, MA, USA), #21103-049), 50% DMEM/F12 (Thermo Fisher Scientific, #11–330-057), 1% penicillin/streptomycin (Merck (Darmstadt, Germany), #A2213), 1% GlutaMax (Thermo Fisher Scientific, #35050-038), 1% B27 supplement (without vitamin A; Thermo Fisher Scientific, #12587-010) and 0.5% N2 supplement (Thermo Fisher Scientific, #17502-048)) supplemented with 0.5 µM PMA (Merck, #540220), 3 µM CHIR (Axon Medchem (Groningen, Netherlands), #Axon1386) and 200 µM ascorbic acid (Sigma-Aldrich (St. Louis, MO, USA), #A4544). For each line, one cryotube of NPCs was thawed, and cells were split into three different 6-wells. The three wells of NPCs were expanded independently and treated as three differentiation replicates. Once NPCs reached 80% confluency, differentiation was started. For the differentiation into human dopaminergic mid-brain neurons (hDaNs), NPCs were incubated in base media supplemented with 20 ng/ml of BDNF (PeproTech (Hamburg, Germany), #450-02), 10 ng/ml of FGF8 (PeproTech, #100-25), 1 µM of PMA and 200 µM of ascorbic acid for 7 days (days 1 to 7). Next, cells were put in base media supplemented with 10 ng/ml of BDNF, 10 ng/ml of GDNF (PeproTech, #450-10), 1 ng/ml of TGF-β3 (PeproTech, #AF-100-36E), 200 µM ascorbic acid, 500 µM dbcAMP (PanReac AppliChem (Darmstadt, Germany), #A0455), and 10 µM DAPT (Selleckchem (Houston, TX, USA), #S2215) for the remainder of the differentiation (days 8 to 23). None of the used media contained fetal calf serum.

### Quantifying the Expression Levels of Neuronal Markers in hDaNs

To quantify the expression of neuronal and dopaminergic markers in the hDaNs, RT-qPCR was performed. Cellular RNA was isolated on day 23 of differentiation using the miRNeasy Tissue/Cells Advanced Mini Kit following the vendor’s instructions (QIAGEN (Hilden, Germany), #217604). The mRNA expression levels of the following genes were quantified: dopaminergic marker tyrosine hydroxylase (TH), mature neuron marker microtubule associated protein 2 (MAP2), midbrain marker forkhead box protein A2 (FOXA2), and LRRK2. RT-qPCR was carried out using the QuantiTect® SYBR®-Green RT-PCR Kit (QIAGEN, #204243, customized primer sequences are listed in Supplemental Table 1). GAPDH was used for internal normalization while RNA isolated from gene-corrected iPSCs derived from L2 was used as an external reference. RT-qPCR was performed on the LightCycler® 480 (Roche) and c(t)-values were extracted using the LightCycler® 480 software (version 1.5.1). The delta-delta-c(t) method was used to calculate the fold change (fc) expression levels of hDaNs over iPSCs.

### Immunocytochemistry of hDaNs

The hDaNs were split on day 8 of the differentiation process and seeded onto 12 mm-wide coverslips. Before seeding, cover slips were coated with 15 µg/ml of Poly-DL-ornithine (Sigma-Aldrich, #P8638) at 37° C for 24 h, followed by coating with 5 µg/ml laminin (Sigma-Aldrich, #L2020) at 37 °C for 4 h. On day 23, cells were fixed by adding 4% PFA to the cells for 20 min. Cells were washed with PBS (Sigma-Aldrich, #D8537) three times before blocking, using PBS containing 10% normal goat serum (NGS) and 0.1% Triton-X for one hour. Blocking buffer was removed, and primary antibodies were added, resuspended in 5% NGS and 0.1% Triton-X in PBS. MAP2 antibody was used in a 1:2000 dilution (abcam (Cambridge, UK), #ab5392) and Tyrosine Hydroxylase (TH) antibody was added in a 1:1000 dilution (Pel-Freez (Rogers, AR, USA), #P40101-150). Primary antibodies were incubated overnight at 4 °C before cells were washed three times with PBS. Secondary antibodies were added in 5% NGS and 0.1% Triton-X. Anti-Chicken AlexaFluor-647 (Thermo Fischer Scientific, #A21449) and Anti-Rabbit AlexaFluor-488 (Thermo Fischer Scientific, #A11070) were used in a dilution of 1:1000. After one hour of incubation at 4 °C, cells were washed three times, and nuclei were stained with Hoechst (Molecular Devices (Urstein, Austria), #H3569) for 5 min. Finally, coverslips were mounted using Dako mounting medium (Aligent (Santa Clara, CA, USA), #S3023). Ten positions were imaged per differentiation and line on a Zeiss Imager.Z1 using the ZEN software (blue edition, Zeiss, (Oberkochen, Germany)). Prior to analysis, Z-stacks were projected, and brightness was adjusted for each channel. The total number of intact nuclei was counted, and the percentage of TH and MAP2-positive cells was calculated.

### Isolation of Extracellular Vesicles from Cell Culture Media

Starting on day 14 of the differentiation protocol, conditioned cell culture media (CCM) was collected from hDaNs every 3 days in order to isolate extracellular vesicles. The CCM was centrifuged at 300 g for 10 min to deplete cell debris before the supernatant was filtered through a 0.22-µm Steriflip filter (Merck, #SCGP00525). The CCM was then concentrated to 1 ml using Amicon® Ultracel Centrifugal filters (Merck, #UFC901024) and centrifugation at 2000*g* at 4 °C for 30 min. Next, the concentrate was recovered and transferred to a 1.5-ml tube. Subsequently, 500µl of Total Exosome Isolation Reagent (Thermo Fischer Scientific, #4478359) was added to 1 ml of concentrated CCM. The samples were then incubated overnight and centrifuged at 10,000*g* for 1 h. The supernatant was discarded, and tubes were centrifuged for 5 min at 10,000*g*. Next, pellets were washed three times with 1 ml PBS before resuspension in 200 µl of PBS containing cOmplete protease inhibitor (Sigma-Aldrich, #11873580001) and phosphatase inhibitor (Sigma-Aldrich, #4906837001).

### Western Blotting of LRRK2 and Extracellular Vesicle Markers

For cell protein extracts, hDaNs from a 6-well were lysed on day 23 of differentiation in 200 µl ice-cold lysis buffer (1% Triton X-100 in PBS) and incubated on ice for 5 min. Resulting cell suspensions were consequently centrifuged at 13,000*g* at 4 °C for 15 min. As for Western blot analysis, 25 to 75 µg of the cell lysates were mixed with 2 × Laemmli sample buffer (Sigma-Aldrich, #S3401-10VL), separated in an 8–10% polyacrylamide gel, and transferred to a 0.45 µm Immobilon-P PVDF membrane (Merck, # IPVH00010). The membranes were blocked in 5% (w/v) milk (PanReac AppliChem, #A0830) in TBS-T for 1 h at RT, incubated with anti-LRRK2 (dilution: 1:500, abcam, #ab133474) or anti-ß-actin (1:5000, abcam, #ab6276) antibodies overnight at 4 °C and with a secondary HRP-conjugated antibody (anti-rabbit: 1:5000, Cell Signalling (Cambridge, UK), #7074; anti-mouse: 1:10 000, Bio-Rad (Hercules, California, USA), #172–1011) for 1 h at RT. For detection, the membranes were incubated with ECL prime Western blot detection reagents (Merck, #GERPN2232), and the signal was detected using Amersham Hyperfilm ECL (Thermo Fisher Scientific, # 10534205). For quantification, the chemiluminescence signal intensity was measured using ImageJ software, and LRRK2 protein levels were normalized to levels of ß-actin expression. For the detection of EV markers, slight modifications were made. Briefly, EV samples were incubated in SDS sample buffer (0.375 M Tris-Cl, 12% SDS, 60% Glycerol, 0.6 M DTT, pH 6.8) for 10 min at 95 °C, run on a 10% polyacrylamide gel, and transferred onto a nitrocellulose membrane using the iblot 2 Dry Blotting System. Anti-Alix (1:250, NovusBio (Littleton, Colorado, USA), #NBP1-90201), anti-Flotillin-1 (1:1000, Cell Signalling, #18634) and anti-CD81 (1:1000, Santa Cruz (Dallas, Texas, USA), sc-166029) antibodies were used to detect corresponding EV-specific markers. Anti-GM130 antibody (1:1000, Cell Signaling, #12480) was used as a negative control. As a positive detection control, 10 µg of neuronal cell lysates were used.

### Nanoparticle Tracking Analysis

Nanoparticle Tracking Analysis (NTA) was used to measure particle size and concentration. The Nanosight NS300 and NanoSight NTA 3.0 0068 software from Malvern Panalytical in Kassel, Germany, were used. For optimal performance, samples were diluted 1:100 to 1:500 in PBS prior to measurement. Each sample was recorded and measured five times.

### Cryo Transmission Electron Microscopy

Cryo Transmission Electron Microscopy (TEM) of the EV samples was conducted in the Nanoscale and Microscale Research Centre (nmRC) of the University of Nottingham, UK. The nmRC has previously published its protocol for preparing EV samples for cryo-TEM, and the protocol was adapted for this manuscript [35]. Holey carbon TEM grids (EM resolutions, Sheffield, UK, #HC300Cu) were used. The EV samples were left to adsorb onto the grids (5 µL/grid) for 2 min; then, excess solution was removed using filters. After blotting the EV samples for a second, they were frozen in liquid ethane using a Gatan CP3 plunge freezing unit (Ametek, Leicester, UK). Frozen samples were loaded onto a FEI Tecnai G2 12 Bio-twin TEM, and Cryo-TEM was completed with an accelerating voltage of 100 kV. Images were obtained using an in-built Gatan SIS Megaview-IV digital camera.

### Quantification of Phosphorylated Rab10 in Cell Lysates and Extracellular Vesicles

Cell lysates as well as EV samples were reduced and alkylated by adding 5 mM DTT and incubated at 56 °C for 30 min followed by alkylation by adding 20 mM Iodoacetamide and incubation in the dark for 30 min. Samples were then resolved on a NuPage 10% Bis-Tris gel (ThermoFischer Scientific, NP0301BOX) Bands containing proteins with a size of 20–30 kDa were excised for In-gel digestion [36]. Briefly, bands were cut into 1 mm gel pieces and then destained by adding 250 µL of destaining buffer (40 mM Ammonium bicarbonate in 40% (vol/vol) Acetonitrile in milli-Q water) and incubated on a Thermomixer for 20 min with agitation at 1200 rpm at room temperature. Following this, the buffer was discarded and this step was repeated to ensure complete destaining. Further, gel pieces were dehydrated by adding 250 µL of 100% (vol/vol) Acetonitrile and incubated on a Thermomixer for 20 min with agitation at 1200 rpm at room temperature; this step was repeated until the gel pieces were completely dehydrated. 500 ng of Trypsin was added in 200µL of trypsin buffer (0.5% (wt/vol) sodium deoxycholate in 20 mM TEABC pH 8.0 in milli-Q water). The tryptic digestion was continued overnight by placing the samples on a Thermomixer at 37 °C while agitating at 1200 rpm. Peptide extraction was performed by adding 100 µL of 99% Isopropanol (vol/vol) in 1% TFA and incubated on a Thermomixer at room temperature with agitation at 1200 rpm for 20 min. The supernatant was transferred to a new 1.5 ml protein lo-binding tube and the extraction was continued another two times; the eluates were pooled and directly loaded on Stage-tips for SDB-RP clean up and the eluted peptides were subjected to vacuum dryness on a SpeedVac concentrator. The peptides were then stored in a −80 °C freezer until LC–MS/MS analysis. For this, peptides were dissolved in LC-buffer (3% ACN vol/vol in 0.1% formic acid vol/vol in milli-Q water) and were spiked with an equimolar ratio of 25 fmol heavy peptide mix containing heavy pRab10 (FHTITp**T**SYYR*), heavy non-phospho Rab10 (FHTITTSYYR*) and two total Rab10 peptides (NIDEHANEDVER* and AFLTLAEDILR*). The peptide mixture was then loaded on Evotips for targeted mass spectrometry analysis as described in PMID: 34125248. The data were acquired in PRM mode on an Orbitrap Exploris 240 mass spectrometer interfaced with an EvoSep LC system. The mass spectrometry data were processed using Skyline software suite for peak picking and the ratio of light/heavy for pRab10, and non-phospho total Rab10 was used in measuring the stoichiometry of phosphorylation [36, 37]. Finally, pRab10 to total Rab10 ratios were normalized to the LRRK2/ß-Actin ratio and values were logarithmized.

### Quantification of Cellular α-Synuclein Levels

Protein expression analysis was performed using the Jess automated capillary western blot system (ProteinSimple). Cell pellets from five independent differentiations were processed on day 18 of the differentiation by double lysis in buffer containing protease and phosphatase inhibitors, followed by adjustment to 1% SDS and sequential centrifugations (1000*g*, 4 °C; then 20,000*g*, RT). Protein concentrations were determined by BCA assay. Each sample was loaded in duplicate to account for technical variability. Samples were mixed with 5 × fluorescent master mix, denatured at 95 °C for 5 min, and loaded (5 μl per capillary) together with a biotinylated molecular weight ladder. Separation was performed at 375 V for 30 min, with increased sample pickup time (12 s) and extended primary antibody incubation (60 min). α-Synuclein was detected using the anti-aSyn MJFR1 antibody (rabbit, Abcam, ab209538), while β-Actin was probed using a mouse monoclonal antibody (R&D Systems, MAB8929) at 1:500. Detection employed an anti-mouse IR secondary antibody (Biotechne, 043–822) and streptavidin-NIR for the ladder (Biotechne, 043–816). Chemiluminescence and NIR fluorescence signals were quantified using the Dropped Lines peak fitting method. Normalization was performed to β-actin.

### Generation of Small-RNA Libraries Using Cell-Free RNA from hDaNs

For the generation of small-RNA libraries, RNA was isolated directly from EVs derived from supernatant from either day 14 to 23 (L1 lines) or day 14 to 17 (L2 lines). RNA was isolated using the miRNeasy Serum/Plasma Advanced Kit (QIAGEN, #217204) and following the vendor’s instructions. After isolation, RNA concentrations were measured using the QubitTM microRNA Assay Kit (Thermo Fischer Scientific, #Q32880) and electropherograms were generated using the high-sensitivity RNA ScreenTape® (Agilent, Santa Clara, California, USA, #5067-5579). For the generation of the small RNA libraries, the SMARTer® smRNA-Seq Kit for Illumina® (Takara (Tokyo, Japan), #635030) was used, and the vendor’s instructions were followed with slight modifications. Briefly, 5 ng of RNA were used for the initial polyadenylation step. This was followed by cDNA synthesis using PrimeScript RT. Finally, cDNA was used for amplification via PCR using one forward primer and differently barcoded reverse primers. The libraries were then bead-purified by first incubating AMPURE beads (Beckmann Coulter (Brea, California, USA), #A63881) with the samples at room temperature for 5 min in a sample-to-bead ratio of 1:1.8. The beads were then incubated on a magnetic rack for another three minutes. The supernatant was discarded, and the beads were washed twice with 80% ethanol and dried for 5 min. Next, the beads were resuspended in 32 µl of water and incubated at room temperature for 5 min. Afterwards, the tubes were placed onto the magnetic rack, and the supernatant was transferred to a new tube. For size exclusion, the same protocol was repeated using SPRI beads (Beckmann Coulter, #B23318) and a sample-to-beads ratio of 1:1. For the generation of electropherograms of the libraries, the high-sensitivity D1000 ScreenTape® (Agilent, # 5067-5584) was used. Finally, libraries were sequenced on a Nextseq550 mid-output, 150 cycles v2.5 flowcell spiking in 30% PhiX control v.3. Although the analysis of mRNA is not part of the present study, sequencing was done at a higher length than usual for small RNA sequencing to allow the inclusion and better mapping of potentially present mRNAs.

### Differential Expression Analysis of Library Sequencing Data

The raw sequence data was transferred to the Quantitative Biology Centre (QBiC), Tübingen, Germany, for geo-redundant long-term storage, data management, and bioinformatics analysis. For data processing, the Nextflow-based nf-core/smrnaseq pipeline (v. 1.2.0) development branch was forked (https://github.com/qbic-projects/smrnaseq, commit: 306b024) to allow for the input of custom adapter trimming parameters. The nf-core/smrnaseq includes various bioinformatic tools, such as FastQC (v0.11.9) (Andrews, 2010) to determine the quality of the FASTQ files. This was followed by read length selection (maximum 40 bp) and adapter trimming using Trim Galore (v0.6.h) [38], based on the recommended parameters by the Takara SMARTer® smRNA-Seq Kit. Subsequently, the filtered reads were mapped to the miRBase [39] database of mature and hairpin human miRNAs using Bowtie1 (v. 1.3.0) [40]. The miRNA-seq data quality was assessed using mirTrace (v1.0.1) [41], followed by an aggregation of the quality control of the analyses with MultiQC (version 1.7; http://multiqc.info/) [42]. Differential expression analysis on the miRNA sequences was then performed using the read counts mapped by Bowtie1. The analysis was performed in R (v3.5.1) using DESeq2 (v1.22.1) [43], through a fork of the Nextflow-based qbic-pipelines/rnadeseq pipeline (v1.32). This fork (https://github.com/qbic-projects/QSCNN, commit: c1aded5) was created to account for the lower number of expressed miRNAs (hairpin, mature) in comparison to typical gene expression data; hence, the variance stabilizing transformation parameter (default: 1000) was adjusted to 400. The linear model employed to model gene expression in DESeq2 was “~Patient+condition_genotype” to account for differences in patients while checking for the effect of the main experimental factor “condition_genotype” (G2019S (mutation in LRRK2) vs. gene-corrected control). The miRNA sequences were considered differentially expressed when the Benjamini–Hochberg multiple testing adjusted *p*-value [44] was smaller than 0.05 (*p*adj < 0.05). Multiple testing corrections were used to minimize the number of false positives.

### Relative Quantification of miRNAs in Cellular and Cell-Free RNA from hDaNs via RT-qPCR

For validation of the differentially expressed miRNAs found in the libraries, a new batch of cell culture-derived EVs was generated, and cell-free RNA was isolated as described previously. For optimal comparison, EVs were isolated from supernatant collected from days 14 through 23 for both the L1 and L2 lines. Cellular RNA was isolated from hDaNs on day 23 of the differentiation. For quantification of specific miRNAs, RT-qPCR was performed using the miRCURY LNA SYBR Green PCR Kit (QIAGEN, #339345) and the vendor’s instructions were followed with slight modifications. Briefly, for reverse transcription, 2 µl of 5 × miRCURY RT Reaction Buffer were mixed with 1 µl of 10 × RT Enzyme Mix and 7 µl of cell-free RNA or 10 ng of cellular RNA. The sample was incubated for one hour at 42 °C, followed by an inactivation step at 95 °C for 5 min. Cell-free cDNA was diluted at 1:30, while cellular cDNA was diluted at 1:60 using nuclease-free water. For the RT-qPCR reaction, 5 µl of 2 × miRCURY SYBR Green Master Mix was added to 1 µl of miRCURY LNA miRNA PCR Assay (QIAGEN, #339306) and 4 µl of diluted cDNA template. Three technical replicates per sample and target were measured. Samples were analyzed in a LightCycler® 480 after an initial PCR heat activation step for 2 min at 95°, followed by 2-step cycling. After 45 runs, melting curve analysis was performed using the LightCycler® 480 Software version 1.5.1, and targets with unspecific amplifications (*c*(*t*) ≥ 40) were excluded. For the extraction of *c*(*t*)-values, LinRegPCR version 2021.2 was used [45]. The delta-delta-c(t) method was used to calculate the relative expression of miRNAs and miRNA-16 was used as an internal reference. miRNAs with a fc of ≥ 1.5 or ≤ 0.5 were considered differentially expressed. A miRNA was considered validated if the direction of dysregulation matched the observation in the library.

### Identification of Protein Targets and Gene Ontology Enrichment Analyses

To identify the predicted protein targets of the miRNAs that were validated as differentially expressed in our study, we used the miRTarBase database [46], considering only those targets supported by strong experimental evidence. Subsequently, we conducted Gene Ontology (GO) enrichment analysis using the enrichGO function from the ClusterProfiler package [47]. A *p*-value threshold of 0.05 was applied, and multiple testing correction was performed using the Benjamini–Hochberg method [44]. Only adjusted *p*-values are reported. To focus on CNS-related GO terms, we filtered for those terms using the grep function in R and a custom list of CNS-associated keywords (Supplemental Table 2). Where applicable, the top 15 significantly enriched GO terms (ranked by *p*-value) were visualized in a semantic scatter plot. A maximum similarity matrix was calculated to highlight the semantic relationships between the identified terms. To further assess the interactome of miRNAs and proteins in our hDaNs, we used a previously published and publicly available proteomic dataset generated using cell lysates from the L1 lines and looked for dysregulated proteins targeted by any of the identified miRNAs [48].

### Collection and Storage of CSF from Study Participants

To validate our in vitro findings, relative expression levels of miRNAs validated in cell culture were quantified in CSF from five PD patients carrying the LRRK2 G2019S mutation, one sPD patient and five healthy controls. Patient L1 had previously provided skin biopsies to generate the iPSC lines used in this study and their CSF was included in this cohort. Control CSF was derived from healthy individuals without any neurological disorders. CSF was collected from individuals following standard protocols. Material was transported from the clinics to the biobank of the Hertie Institute and processed within 30min after withdrawal. CSF was centrifuged at room temperature for 10 min at 2000*g*. Each sample was then transferred to one 15-ml falcon tube and homogenized by vortexing before being aliquoted into cryotubes. The CSF was frozen and stored in the biobank at −80 °C. Prior to RNA isolation, CSF samples were slowly thawed on ice.

### Relative Quantification of miRNAs in Patient CSF

Cell-free RNA was isolated from 400 µl of CSF using the miRNeasy Serum/Plasma Advanced Kit. Briefly, 120 µl RPL solution was added to the CSF, followed by 40 µl of RPP reagent. The subsequent steps were then carried out according to the vendor’s instructions. RT-qPCR was performed as described previously using commercially available primer sets from QIAGEN, and *c*(*t*)-values were extracted using the LinRegPCR software. miRNA-16 was again used as a reference, and the results were expressed as the fc of the expression levels in CSF from the matching healthy control.

### Data Analysis

Statistical analysis, except for the analysis of the small RNA-Seq data, was performed using either GraphPad Prism software, version 9.3.0. (La Jolla, CA, United States) or R-Studio, version 4.3.0. Normal distribution of the data was checked using QQ-plots. Non-parametric data (mRNA expression levels) were log-transformed, the alpha level was set at 0.05, and mean differences and standard deviations are reported. Ordinary two-way ANOVA followed by Šidák’s test was used to test for differences between the genotypes when analyzing the mRNA expression levels and the results of the immunofluorescence staining. Factors were mutation status (LRRK2 G2019S and LRRK2 wildtype) and target (ICC: MAP2 and TH; mRNA expression: MAP2, TH, FOXA2, LRRK2), L1 and L2 lines were tested separately. For the analysis of LRRK2 expression in cell lysates and pRab10 ratios in cell lysates and EVs, a two-sample *t*-test was performed to compare the two genotypes. Paired *T*-test was used to assess differences in α-Synuclein levels between lines. Individual differentiations are indicated as n_Diff_. Figures were built using either the GraphPad Prism software, RStudio (version 2022.12.0+353) or Python (version 3.10) [49].

## Results

### Immunocytochemistry of Neuronal and Dopaminergic Marker

**Fig. 1:**
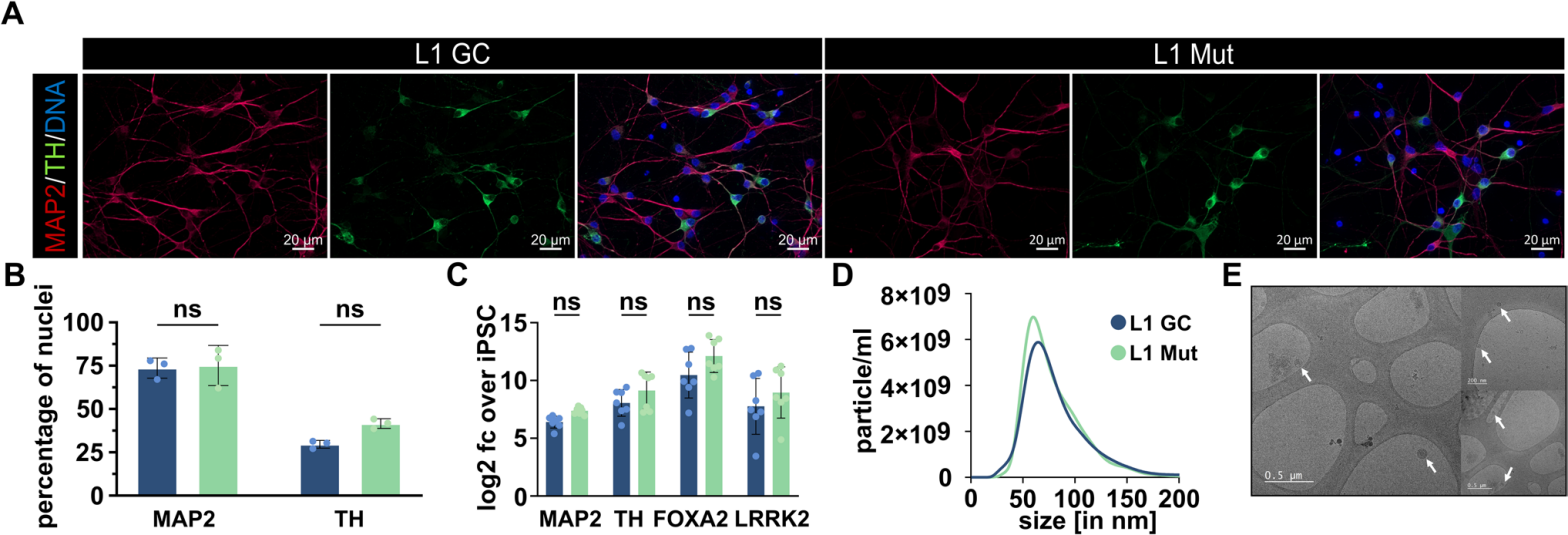
Validation of neuronal identity and extracellular vesicles. **A** Representative images of immunofluorescence staining of hDaNs from the L1 lines on day 23 of the differentiation. Dopaminergic marker TH was stained together with neuronal marker MAP2 and DNA. **B** Percentages of MAP2+ and TH+ cells were calculated for both the L1 lines. 10 different positions were analyzed (*n*_Diff_ = 3). Error bars indicate standard deviation.** C** RT-qPCR was performed on cellular RNA isolated on day 23 from the L1 lines to quantify expression of MAP2, TH, FOXA2, and LRRK2 on mRNA level (*n*_Diff_ = 7). Results are shown as log2 fc of the expression in iPSC control samples. Error bars indicate standard deviation. **D** NTA measurements of EVs derived from both L1 lines. Size ranged between 30 and 200 nm. **E** Representative Cryo-EM images of spherical and membrane-encapsulated vesicles

Immunofluorescence staining of hDaNs revealed the neuronal morphologies of the cells and the presence of the dopaminergic marker TH and the mature neuron marker MAP2 (Fig. 1A). In L1 GC, 73.6% (SD: ± 5.8%) of the cells were MAP2 positive, compared to 75.1% (SD: ± 11.6%) in L1 Mut. Furthermore, 29.6% (SD: ± 2.2%) of the cells in L1 GC and 41.6% (SD: ± 2.8%) of the cells in L1 Mut showed TH expression (Fig. 1B). Ordinary two-way ANOVA was performed to test for differences in percentages of TH or MAP2 positive cells between the two genotypes, and no significant interaction was observed between target and genotype (*F*(1, 8) = 1.81, *p* = 0.215). Simple main effects analysis showed that target (*F*(1, 8) = 99.72, *p* < 0.0001) had a significant effect on the percentage of positively expressing cells but not genotype (*F*(1, 8) = 2.971, *p* = 0.123). After correction for multiple comparisons using Šidák’s test, neither the difference of TH positive cells (mean difference: 0.12 percentage points (p.p.), *q* = 3.07, DF = 8, *p* = 0.211) nor of MAP2 positive cells (mean difference: 0.01 p.p., *q* = 0.377, DF = 8, *p* = 0.993) was observed to be significantly different between genotypes. Results from the L2 lines can be found in the Supplemental Materials and Methods (Supplemental Fig. 1A and 1B).

### mRNA Expression Levels of Neuronal and Dopaminergic Marker

Our quantification of TH, MAP2, and FOXA2 mRNA in the differentiated lines showed strong upregulation compared to iPSCs (Fig. 1C). As for LRRK2 mRNA levels, two-way ANOVA indicated that there was no statistically significant interaction between the effects of genotype and target in the L1 lines (*F*(3, 48) = 0.120, *p* = 0.948). Simple main effects analysis showed that both genotype and target had a statistically significant effect on the expression levels (target: *F*(3, 48) = 17.90, *p* < 0.0001; genotype: *F*(1, 48) = 7.926, *p* = 0.007). After correction for multiple comparisons using Šidák’s test, no significant difference between L1 Mut and L1 GC was found for any of the other gene expression levels. Results from the L2 lines can be found in the Supplemental Materials and Methods (Supplemental Fig. 1C).

### Confirmation of Presence of Extracellular Vesicles

After validating the neural identity of the cells, we wanted to confirm the successful isolation of EVs from cell culture supernatants. The size of particles from all lines ranged from 30 to 200 nm (measured using NTA, Fig. 1D and Supplemental Fig. 1D). In Cryo-TEM, particles appeared as solitary, spherical, and membrane-encapsulated structures, as previously reported (Fig. 1E) [50]. The mean size of particles isolated from L1 GC was 80.53 nm (SD: 6.89, n_Diff_ = 6) and 77.86 nm (SD: 5.05, n_Diff_ = 6) in L1 Mut. Furthermore, we tested for the presence of EV protein markers. The cytoplasmic protein Alix and the membrane-bound proteins Flotillin-1 and CD81 were detected in Western blots, while GM130 was barely detectable (Supplemental Fig. 1E) [51]. Taken together, particle size, the presence of EV protein markers and Cryo-TEM images confirmed the presence of EVs, following previously published guidelines [52]. Results from the L2 lines can be found in the Supplemental Materials and Methods.

### Differential Expression of LRRK2 and pRab10

**Fig. 2:**
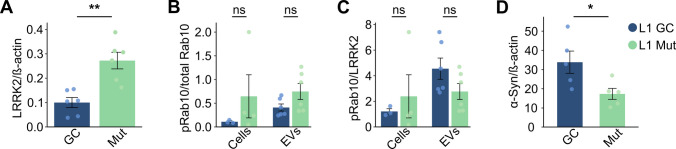
Differential expression of LRRK2, pRab10, and α-synuclein. **A** Quantitative analysis of LRRK2/β-Actin ratios revealed a significant increase in LRRK2 expression in the L1 Mut line compared to L1 GC. **B** We measured pRab10 to total Rab10 ratios in both cell lysates and EVs, with each data point representing an independent replicate. While overall kinase activity did not differ significantly between genotypes in either matrix, pRab10 levels in EVs from the mutant line exhibited a non-significant trend toward elevation (*p* = 0.096). **C** Bar charts show pRab10/tRab10 ratios now normalized to the corresponding LRRK2/β-Actin ratio. This normalization was performed to determine whether any genotype‐dependent effects would become more pronounced when adjusted for the cellular LRRK2 expression level. However, no statistically significant differences were detected. **D** Lastly, hDaNs were characterized for differences in α-synuclein expression using the JESS system. Paired *t*-test revealed a significant decrease in α-synuclein expression in the mutant line after normalization to ß-actin (*p* = 0.035)

To fully characterize our cell lines, we next assessed LRRK2 protein expression via Western blotting (Supplemental Fig. 2A). In the comparison of log2-transformed LRRK2 expression between genotypes within the L1 cell lines, an unpaired two-sample *t*-test revealed significantly higher LRRK2 expression in G2019S mutant cells compared to gene-corrected controls (*t*(10) = −4.32, *p* = 0.0015) (Fig. 2A). In the L2 mutant line, LRRK2 was hardly detectable and the gene‐corrected controls showed significantly higher expression (*t*(4) = 8.96, *p* < 0.001) (Supplemental Fig. 1F and 1G). As pointed out above, given the markedly reduced LRRK2 protein in the L2 mutant line, we elected to focus our analyses on the L1 lines for the remainder of this study.

To assess LRRK2 kinase activity, we compared pRab10/tRab10, as well as pRab10/tRab10 ratios normalized to LRRK2 expression (pRab10/LRRK2), in both cell lysates and EVs. In cell lysates from the L1 lines, pRab10/tRab10 ratios did not differ significantly between genotypes (*t*(5) = −1.00, *p* = 0.36) (Fig. 2B). Similarly, normalization to LRRK2 levels did not reveal a significant difference (*t*(5) = −0.59, *p* = 0.58) (Fig. 2B). In EVs derived from the L1 lines, the difference in pRab10/tRab10 ratios showed a trend toward higher values in the mutant line, though it did not reach statistical significance (*t*(10) = −1.84, *p* = 0.096) (Fig. 2B). Normalized pRab10/LRRK2 ratios also did not differ significantly between genotypes (*t*(10) = 1.72, *p* = 0.12) (Fig. 2C). Results from the L2 lines can be found in the Supplemental Materials and Methods (Supplemental Fig. 1H and 1I).

### Differential Expression of α-Synuclein

Finally, as α-synuclein accumulation is a hallmark of PD pathology, we also examined α-synuclein expression levels in cell lysates from the L1 lines to explore potential genotype-dependent differences (Supplemental Fig. 2B). We measured α-synuclein/β-actin ratios and performed a paired *t*-test comparing the mutant and isogenic control lines. This analysis revealed a statistically significant decrease in α-synuclein levels in the mutant line (*t*(4) = 3.14, *p* = 0.035) (Fig. 2D).

### Quality Control of Small RNA Libraries

Notably, RNA input from exosomes did not contain typical rRNA peaks that are usually used to verify RNA integrity (Supplemental Fig. 3). This has previously been reported to be the case for exosomal RNA[17]. The mean GC percentage of the reads was 55.17% (SD: ± 4.47), while the mean percentage of duplicate reads was 53.39% (SD: ± 9.71). The mean percentage of miRNAs present in the libraries was 2.36% (SD: ± 1.71). The percentage of reads that are mapped to miRBASE is 1.78% (SD: ± 1.01) for mature RNAs and 3.38% (SD: ± 1.75) for hairpin RNAs. These quality control values and statistics are generally in line with previously published small RNA libraries [53]. Further details (including the proportion of biotypes) are reported in Supplemental Fig. 4 and Supplemental Tables 3–6.

### Small-RNA Libraries Reveal a Subset of Differentially Expressed miRNAs

**Fig. 3:**
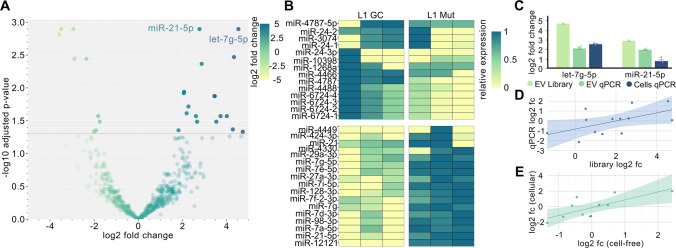
Results of the small-RNA-libraries and validation RT-qPCR. **A** A total of 2611 miRNAs were detected in the L1 libraries. 31 miRNAs passed the threshold and were considered differentially expressed. Log2 fc is expressed as a change of L1 Mut over L1 GC. The horizontal line indicates the *p*-value threshold. For visualization purposes, the *y*-axis is capped at the 99th percentile to prevent extreme outliers from stretching the scale. **B** The heatmap visualizes the 31 miRNAs that were significantly differentially expressed. vst-transformed gene counts were normalized from 0 to 1 for each target, with 0 corresponding to the lowest observed expression and 1 to the highest. Colors reflect each sample’s relative expression level for a given miRNA. **C** RT-qPCR was conducted using both cell-free and cellular RNA. In L1, the dysregulation of let-7g-5p and miR-21-5p was successfully validated. **D** Pearson’s correlation analysis revealed a strong directional correlation between miRNA log2 fold change levels measured in EV-derived RNA libraries and RT-qPCR (*R*^2^ = 0.301, *p* = 0.052). **E** Additionally, we conducted a correlation analysis of the log2 fold changes in miRNA expression between cellular and cell-free RNA. This analysis included data from miRNAs whose expression changes observed in the libraries could not be validated. We identified a significant correlation between the two compartments, indicating that alterations in cell-free miRNA expression reflect those in the cellular miRNAome

In the L1 libraries, a total of 2611 miRNAs were detected (Fig. 3A). Of these, 798 did not meet the fold-change threshold and were excluded from further analysis. After correction for multiple testing, 19 miRNAs were significantly upregulated and 12 were significantly downregulated in the L1 mutant line compared to the gene-corrected control (Fig. 3B, Supplemental Table 7). Results from the L2 libraries can be found in the Supplemental Materials and Methods (Supplemental Fig. 5 and Supplemental Table 8).

### Validation of Differentially Expressed miRNAs

After assessing the technical variation of each miRNA across replicates within the libraries, we selected seven miRNAs with low technical variability for RT-qPCR quantification in each patient line (Supplemental Table 9). To validate the library-based quantifications, miRNAs identified as differentially expressed in one patient line were additionally measured in the other line, allowing us to more accurately assess the correlation of library and qPCR-based quantification. A new and independent batch of EVs was generated and cell-free RNA was isolated and used for this validation step. Of the seven miRNAs we identified in the L1 libraries and tested in the new batch, two miRNAs could be validated in the L1 lines (log2 fc value: let-7g-5p: 1.99; miR-21-5p: 1.84) (Fig. 3C). Table 1 summarizes the results from the RT-qPCR experiment using cell-free RNA derived from the L1 lines. Next, we plotted the respective log2 fc values from the libraries against the log2 fc from the RT-qPCR experiments to assess the reliability of library quantifications. Using Pearson’s correlation, we observed a moderate positive relationship between library fold changes and RT-qPCR measurements in material derived from the L1 lines, that fell just short of statistical significance (*r*(11) = 0.55, *p* = 0.052, *R*^2^ = 0.30) (Fig. 3D). Results from the L2 validation qPCRs can be found in the Supplemental Materials and Methods (Supplemental Fig. 5 and Supplemental Table 10).

**Table 1:** Overview of the validation RT-qPCRs in cell-free RNA. Displayed miRNAs were identified in the L1 libraries. Targets where dysregulation crossed the fc threshold of either ≥ 1.5 or ≤ 0.5 over the gene corrected control are highlighted in bold. An * highlights those miRNAs, where dysregulation found in the library was confirmed

Target	fc (L1)
miR-21-5p*	**3.58 (SD ± 0.21)**
let-7g-5p*	**4.00 (SD ± 0.59)**
miR-128-3p	**0.42 (SD ± 0.07)**
miR-29a-3p	1.19 (SD ± 0.14)
miR-27a-3p	1.17 (SD ± 0.24)
miR-24-3p	1.15 (SD ± 0.28)
miR-424-3p	1.06 (SD ± 0.22)

### Differentially Expressed Cell-Free RNAs Are Indicative of Changes in the Cellular miRNAome

We proceeded to quantify the expression levels of the miRNAs selected from our libraries using cellular RNA. Interestingly, the direction of dysregulation was mostly identical between cellular and cell-free RNA, including the two miRNAs we could validate from the libraries in cell-free RNA (Fig. 3C). Results of the RT-qPCR using cellular RNA are summarized in Table 2. To further analyze the relation between cellular and cell-free RNA in the L1 line, we performed Pearson correlation analysis using fold change values. A significant positive correlation was observed between cellular and cell-free RNA in L1 (*r*(9) = 0.75, *p* = 0.008, *R*^2^ = 0.56), suggesting concordant deregulation of miRNAs across compartments (Fig. 3E). Results from the L2 lines can be found in the Supplemental Materials and Methods (Supplemental Fig. 5 and Supplemental Table 11).

**Table 2:** Overview of the RT-qPCRs results using cellular RNA. Displayed miRNAs were identified in the L1 libraries. Targets where dysregulation crossed the threshold of either ≥ 1.5 or ≤ 0.5 over the gene corrected control are highlighted in bold

Target	fc (L1)
miR-21-5p	**1.62 (SD ± 0.32)**
let-7g-5p	**5.37 (SD ± 0.32)**
miR-128-3p	0.91 (SD ± 0.03)
miR-27a-3p	1.17 (SD ± 0.08)
miR-24-3p	1.40 (SD ± 0.09)

### Functional Enrichment and Proteomic Validation of miRNA Target Proteins

**Fig. 4:**
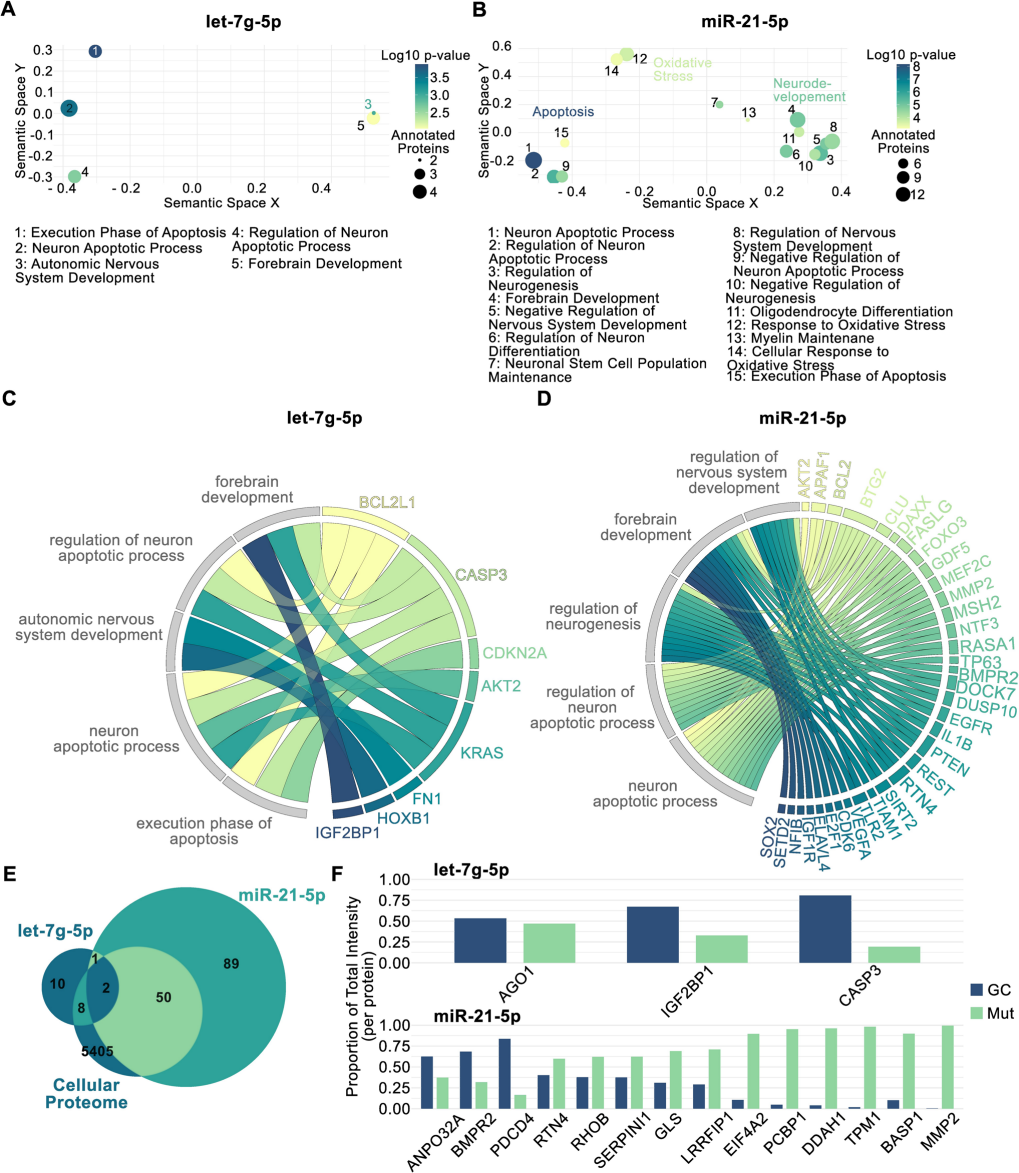
Potential protein interactions mediated by let-7g-5p and miR-21-5p. **A** The semantic scatter plot displays GO terms significantly associated with the proteome targeted by let-7g-5p or **B** miR-21-5p. Terms were filtered for their association with the CNS and then arranged in a two-dimensional space according to their semantic similarity. Closely related terms appear nearer to each other; dot size reflects the number of annotated proteins, and color indicates the log₁₀ of the p-value. For let-7g-5p only five terms were identified; for miR-21-5p the top 15 terms as sorted by their p-values are displayed. **C** Chord diagrams show the top five CNS-filtered GO terms for let-7g-5p and **D** miR-21-5p, with each term linked to proteins that are both annotated to that GO category and directly targeted by the respective miRNA. **E** A publicly available proteomic data set generated for the L1 lines was used to assess expression changes in proteins targeted by each miRNA. Of the 21 proteins targeted by let-7g-5p, 10 were within this data set. For miR-21-5p, of the 142 proteins targeted by this miRNA, a total of 52 were within the proteomic data set. One protein was targeted by both miRNAs but not within the proteomics data set. **F** We evaluated differential expression for all proteins both targeted by the miRNAs and present in the proteomic dataset. Of these, three let-7g-5p targets and fourteen miR-21-5p targets showed significant expression changes

Using the mirTarBase database, we identified 21 proteins targeted by let-7g-5p and 142 targeted by mir-21-5p (Supplemental Table 12). Functional enrichment analysis was performed for each miRNA separately, focusing on the biological processes significantly associated with the predicted target proteins. Among the significantly associated GO terms for let-7g-5p were the *execution phase of apoptosis* and the r*egulation of* the* neuron apoptotic process* (Fig. 4A, Supplemental Table 13). Proteins targeted by miR-21-5p were significantly associated with terms related to *neuronal apoptosis*, *oxidative stress* or *neurodevelopment* (Fig. 4B, Supplemental Table 14). The top five CNS-filtered GO terms for let-7g-5p and miR-21-5p, with each term linked to proteins that are both annotated to that GO category and directly targeted by the respective miRNA are depicted in Fig. 4C, D.

Using a previously published proteomic dataset of L1 hDaN cell lysates, we identified 52 proteins predicted to be targets of miR-21-5p and 10 proteins targeted by let-7g-5p that were quantifiable within the cellular proteome (Fig. 4E). Among the proteins quantified in the cell lysates derived from L1 hDaNs, three targets of let-7g-5p (AGO1, IGF2BP1, CASP3) and three targets of miR-21-5p (ANP32A, BMPR2, PDCD4) were found to be downregulated in the L1 mutant line. In contrast, 11 proteins predicted to be targeted by miR-21-5p were upregulated in L1 Mut, including RTN4, RHOB, SERPINI1, GLS, LRRFIP1, EIF4A2, PCBP1, DDAH1, TPM1, BASP1, and MMP2 (Fig. 3F).

### PD Patients Carrying the LRRK2 G2019S Mutation Display Dysregulated miRNAs in CSF

**Fig. 5:**
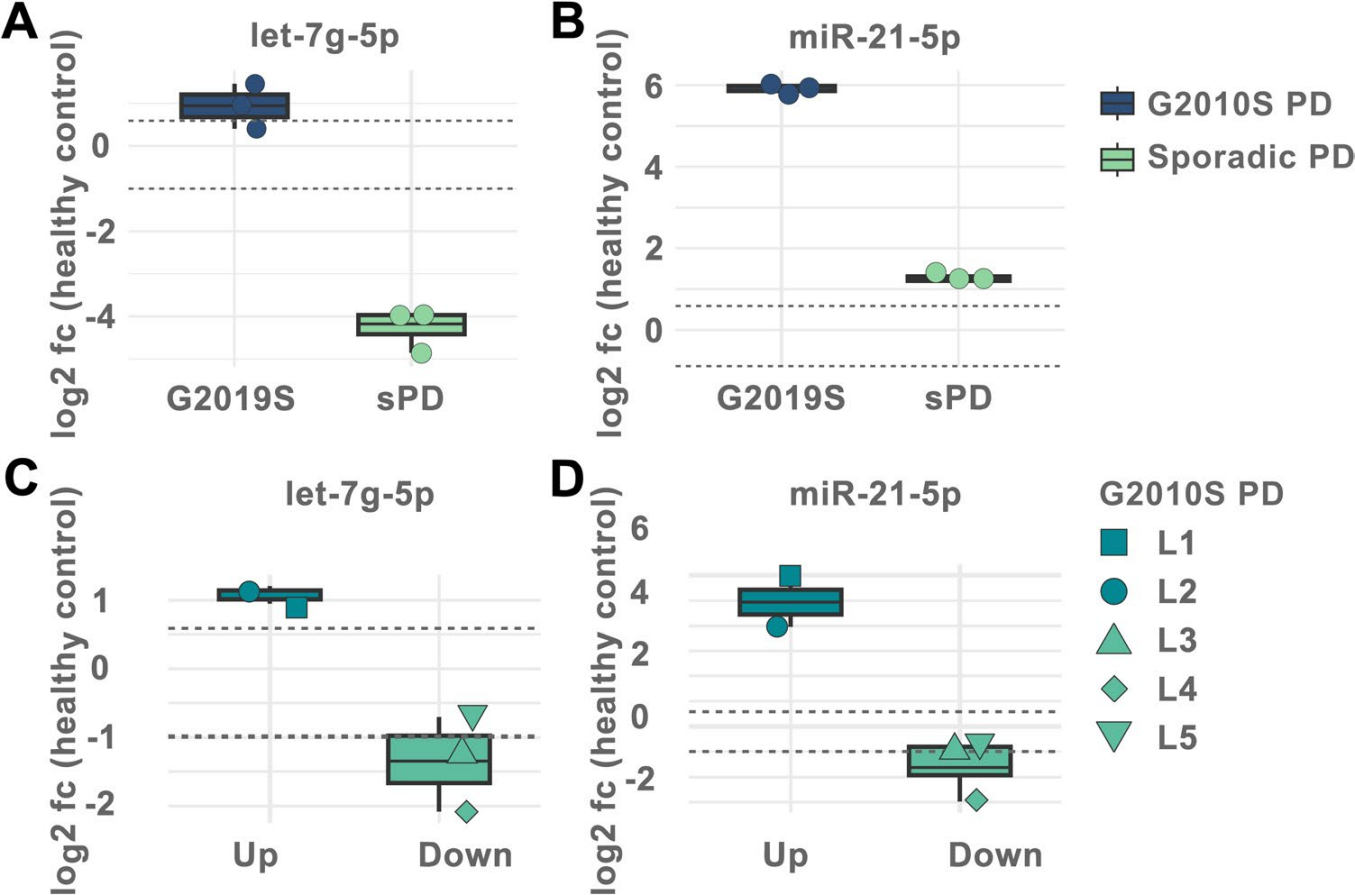
Quantification of let-7g-5p and miR-21-5p in patient-derived cerebrospinal fluid. **A** Expression levels of let-7g-5p and **B** miR-21-5p were quantified in the CSF of a patient with fPD carrying the LRRK2 G2019S mutation, as well as an age- and gender-matched patient with sporadic PD (sPD) and a healthy control. Notably, the fPD patient also served as the fibroblast donor for the NPCs used in the in vitro experiments of this study. Expression changes are expressed as log2 fc over the matched healthy control. Expression levels of the respective miRNAs were elevated in the CSF of the LRRK2 mutation carrier, consistent with the upregulation observed in vitro. In the sPD patient, let-7g-5p levels were decreased, whereas miR-21-5p levels were increased compared to the healthy control, suggesting that these miRNA changes may reflect general PD pathology rather than being specific to LRRK2-associated cases. Each dot represents a technical replicate. Dotted horizontal lines indicate the fc-threshold of ± 1.5. **C** Expression levels of let-7g-5p and **D** miR-21-5p were quantified in CSF from an additional four fPD patients carrying the LRRK2 G2019S mutation, each matched to a healthy control of similar age and gender. In addition to the previously described patient L1, a second LRRK2 carrier showed upregulated expression of both miRNAs. In contrast, the remaining three patients exhibited slightly to moderately reduced levels of both let-7g-5p and miR-21-5p compared to their respective controls. Each data point represents the mean of technical replicates, with shapes indicating the individual patients. Thick line indicates group mean. Dotted horizontal lines again indicate the fc-threshold of ± 1.5

To assess the translational relevance of our in vitro findings, we next evaluated expression levels of the candidate miRNAs in patient-derived CSF. Specifically, we isolated cell-free RNA from the CSF of a patient carrying the *LRRK2* G2019S mutation who had also donated the fibroblasts used to generate the neuronal cells in this study. An age- and gender-matched healthy control was included for comparison. Clinical data can be found in Supplemental Table 15. Expression levels of let-7g-5p and miR-21-5p were quantified using miR-16 as an internal reference (Supplemental Tables 16 and 17). Notably, both miRNAs were found to be upregulated in the patient’s CSF, aligning well with our in vitro observations (Fig. 5A, B). To investigate whether these alterations are specific to LRRK2-associated PD, we extended the analysis to a matched sPD patient. In this case, let-7g-5p levels were decreased, while miR-21-5p was modestly elevated compared to the control. Although based on a single-subject comparison, these preliminary findings suggest that the observed miRNA changes may reflect broader PD-associated molecular alterations rather than being exclusive to LRRK2-linked pathology (Fig. 5A, B).

Finally, we extended our analysis to a larger cohort of fPD patients carrying the LRRK2 G2019S mutation. Including patient L1, we quantified expression levels of let-7g-5p and miR-21-5p in a total of five female fPD patients and their respective age- and gender-matched healthy controls. Detailed clinical information is provided in Supplemental Table 15. In addition to L1, one other patient exhibited upregulation of both miRNAs, whereas the remaining three patients showed moderate downregulation (Fig. 5C, D). Exact fold change values are reported in Supplemental Tables 16 and 17.

## Discussion

In this study, we investigated the impact of the LRRK2 G2019S mutation on miRNA expression profiles in neuronal EVs derived from human dopaminergic neurons. We further explored the potential of cell-free miRNAs as accessible biomarkers for LRRK2-linked PD. Utilizing an iPSC-based in vitro model, we conducted a comprehensive molecular characterization of isogenic mutant and control lines. We identified and validated two differentially expressed miRNAs, namely let-7g-5p and miR-21-5p, as consistently upregulated in the LRRK2 mutant line across independent experimental batches. Both miRNAs were subsequently detected in the CSF of the patient from whom the iPSC line had been derived, as well as in additional LRRK2 G2019S carriers and a sPD case, highlighting their potential relevance beyond the in vitro model. Our findings support the concept that neuronal EV-associated miRNAs can reflect intracellular regulatory processes and may serve as informative markers of PD-related molecular alterations in genetically defined contexts. They further highlight the feasibility of linking patient-derived cellular models to clinically accessible biofluids, thereby enabling individualized validation of candidate biomarkers.

We employed iPSC-derived dopaminergic neurons carrying the LRRK2 G2019S mutation as an in vitro model for fPD. Neuronal identity was confirmed by quantifying the neuronal marker MAP2, which was robustly expressed in the differentiated cells. Additionally, 30–40% of all cells expressed TH, a marker for dopaminergic neurons, in line with previous reports [54, 55]. Expression of the midbrain-specific transcription factor FOXA2 was significantly elevated compared to undifferentiated iPSCs, supporting a midbrain dopaminergic phenotype [56, 57]. A more comprehensive phenotypic characterization of the iPSCs used in this study—including additional dopaminergic markers and dopamine uptake assays—has been published previously [33].

To investigate extracellular RNA, we isolated EVs from the culture medium and confirmed their presence through NTA, Cryo TEM, and detection of canonical EV markers by Western blot [52]. As part of our initial molecular characterization, we assessed LRRK2 expression at the mRNA and protein levels using qPCR and Western blot. Marked differences emerged between the two mutant lines: LRRK2 was upregulated in L1 but strongly downregulated in L2, where protein levels were nearly undetectable. Although the mechanisms regulating LRRK2 expression remain incompletely understood, prior studies have documented variability in its expression across tissues and pathological states. For example, elevated LRRK2 levels have been reported in colonic tissue of PD patients [58] and its expression is known to be modulated by other PD-associated proteins such as PINK1 [59]. The marked differences observed between the two mutant lines may also reflect differentiation-dependent mechanisms, which represent an important consideration for future iPSC-based studies. Due to the pronounced suppression of LRRK2 in the L2 line and the associated difficulty in interpreting downstream effects, in the present study, we focused our analysis on the L1 line.

To further characterize our model, we assessed kinase activity using pRab10/tRab10 ratios in both cell lysates and EVs. While pRab10/tRab10 ratios were comparable between genotypes in cell lysates, EVs showed a non-significant trend toward increased phosphorylation in the mutant line. This mild elevation, although not statistically significant, is consistent with earlier studies reporting subtle increases in LRRK2 kinase activity in G2019S mutation carriers [36]. Together, these findings underline the hypothesis that the pathogenic mutation does not uniformly drive strong kinase hyperactivation but may instead induce more nuanced and compartment-specific changes [60, 61]. Given the complexities of pRab10 as a readout of LRRK2 kinase activity, more detailed investigations will be required to fully resolve its role in disease-related signaling.

Building on the results from the small RNA sequencing libraries, which revealed a distinct pattern of differentially expressed miRNAs in the L1 mutant line compared to the gene-corrected control, we proceeded to select a subset of candidates for further validation. Of the seven miRNAs selected for validation in the L1 lines, let-7g-5p and miR-21-5p, both of which were upregulated in EVs from the L1 Mut line, were successfully validated in an independent experimental batch.

As for the functional relevance of these two miRNAs, intracellular let-7g-5p is regarded as a protective miRNA, owing to its documented ability to repress α-synuclein protein expression [62], and its potential as a target in disease-modifying therapies is currently under active investigation [63]. Moreover, elevated levels of let-7 family members have been reported to attenuate LRRK2-associated pathogenic effects [26]. The observed upregulation of let-7g-5p in the L1 mutant line may therefore represent a compensatory cellular response aimed at mitigating LRRK2-driven dysfunction. On the other hand, let-7 family members might exert neurotoxic effects when released extracellularly. Let-7 carries a motif that activates neuronal Toll-like receptor 7 (TLR-7), acting as a danger-associated molecular pattern and triggering apoptosis [64]. Taken together, let-7g-5p appears to play a dual regulatory role in the context of PD, functioning intracellularly as a protective factor counteracting LRRK2-driven pathogenic mechanisms, while potentially promoting neuroinflammatory damage when released from cells. To date, no studies have simultaneously assessed intracellular and extracellular levels of let-7g-5p in human dopaminergic neurons or in the CSF of PD patients. Our findings therefore provide novel insights into both the cellular regulation and potential extracellular signaling role of this miRNA in the context of LRRK2-linked PD.

miR-21 has been implicated in various central nervous system disorders, including AD and PD, where it is known to regulate both inflammatory pathways and apoptotic signaling [65]. On one hand, miR-21 exerts pro-survival effects by promoting cell viability and proliferation while suppressing apoptosis, which may be neuroprotective in certain contexts [65]. On the other hand, miR-21 might also act as a neuroinflammatory mediator, similar to let-7, via activation of TLR-7 [66]. In addition, elevated miR-21 levels have been associated with increased α-synuclein expression [67] and were observed in the substantia nigra of postmortem brain samples from six PD patients [68]. Again, no previous study has investigated the role of extracellular miR-21-5p in the context of LRRK2-associated PD or PD in general.

As both miR-21-5p and let-7g-5p reportedly play a role in the regulation of α-synuclein expression, we proceeded to quantify cellular α-synuclein in the L1 lines. Here, we observed that α-synuclein levels in the mutant line were slightly lower than in the gene-corrected control. At first glance, this finding may appear counterintuitive, as one might expect elevated α-synuclein expression in a PD model carrying the LRRK2 G2019S mutation. However, when considered in the context of the two dysregulated miRNAs, several biologically meaningful interpretations emerge.

It is conceivable that the presence of the LRRK2 G2019S mutation initiates pathogenic processes that would typically promote an upregulation of α-synuclein expression, one of which could be the dysregulation of miR-21-5p. Upon closer analysis, our data revealed that intracellular miR-21-5p was only modestly elevated (1.6-fold), whereas it was strongly enriched in EVs, with a 3.6-fold increase. This observation may suggest an active cellular mechanism that preferentially packages and secretes miR-21-5p into EVs in an attempt to reduce its intracellular levels and thereby mitigate its potentially dysregulatory effects on α-synuclein expression. In parallel, we observed a marked upregulation of the protective let-7g-5p, both in EVs (4.0-fold) and intracellularly (5.4-fold). This pattern further supports the hypothesis of a coordinated cellular response aimed at compensating for the pathological influence of the LRRK2 mutation. Taken together, these findings raise the possibility that in young neurons—as used in our in vitro model—robust compensatory mechanisms may be able to effectively buffer early pathogenic triggers, including those that would otherwise elevate α-synuclein. As PD is a neurodegenerative disorder primarily affecting older individuals, one could speculate that the gradual decline in the efficiency of such compensatory systems over time may represent a critical step in disease progression.

To gain further insight into the potential functional consequences of the miRNA dysregulation observed in our model, we performed GO enrichment analysis on predicted protein targets of let-7g-5p and miR-21-5p. Interestingly, GO terms associated with let-7g-5p centered around apoptotic regulation, suggesting a potential neuroprotective role for let-7g-5p through suppression of cell death pathways. This aligns with previous findings reporting its ability to reduce α-synuclein expression and attenuate PD-related toxicity [62, 63]. In contrast, the broader set of proteins targeted by miR-21-5p was enriched for functions beyond apoptosis, including oxidative stress regulation, indicating a more complex or pleiotropic role for this miRNA. Notably, when integrating these findings with protein-level data from our previously published L1 hDaN proteome, several predicted targets were indeed dysregulated in the mutant line. Together, these findings underscore the potential biological relevance of let-7g-5p and miR-21-5p in the context of LRRK2-mutant human dopaminergic neurons.

Upon quantifying both cellular and extracellular miRNA expression levels, we observed a statistically significant correlation between the intracellular and cell-free miRNAome in our in vitro model. While this finding may initially appear self-evident, given that extracellular miRNAs must originate from the cells, it actually addresses the important question of whether changes in the cellular miRNAome driven by PD-specific stressors are faithfully reflected in secreted miRNAs. In fact, there is evidence that miRNA packaging into EVs is selective rather than random and in some cellular models, only subsets of intracellular miRNAs are secreted while others are retained [69, 70]. This highlights that the extracellular presence of a given miRNA might not always just be a reflection of intracellular levels. Studying the relationship between the intra- and extracellular miRNAome is also relevant from a mechanistic perspective. As exemplified by miR-21-5p, while the relative direction of expression change (i.e., upregulation) may in most cases be consistent between intracellular and extracellular compartments, the magnitude of these fold changes can offer important insights into underlying disease-related mechanisms. Our study demonstrates a robust and statistically significant correlation between intra- and extracellular miRNAome profiles in a disease-relevant human iPSC-derived neuronal model of LRRK2-linked PD. These findings support the concept that, at least in this system and model, cell-free miRNAs can serve as a reliable surrogate for intracellular miRNAome dynamics and may hold promise as accessible biomarkers of disease-related molecular processes.

In the final part of our study, we investigated whether the dysregulated miRNAs identified in vitro could also be detected in patient-derived CSF. The CSF of the individual who had donated the fibroblasts used to generate the LRRK2 G2019S iPSC line showed increased levels of both miRNAs, mirroring the findings from the corresponding neuronal cell model. This concordance between extracellular miRNA signatures in vitro and in vivo strengthens the translational relevance of our approach and provides a compelling proof-of-concept for individualized target validation strategies. The observation that both miRNAs were also altered, albeit to varying degrees, in other LRRK2 G2019S mutation carriers underscores their potential role as accessible molecular markers. The divergent profiles may reflect biological heterogeneity among mutation carriers, including differences in disease stage, penetrance, or compensatory molecular responses. The inclusion of a sPD case offered additional perspective on the specificity of the observed miRNA alterations. Both let-7g-5p and miR-21-5p were also dysregulated in this patient’s CSF, albeit with a distinct expression pattern compared to its matching LRRK2 case. While preliminary, these findings suggest that the dysregulation of the identified miRNAs may not be exclusive to LRRK2-linked PD, but could instead reflect broader, mutation-independent disease mechanisms. This interpretation is supported by several previous studies employing both in vitro and in vivo approaches, which likewise reported no major differences in miRNA expression profiles between sporadic PD cases and those carrying a LRRK2 mutation [28, 29]. Extending this approach to additional LRRK2 variants and sporadic PD cases will be essential to further increase the translational relevance of the findings.

## Limitations

While our findings provide preliminary evidence that in vitro results obtained from patient-derived iPSC models can be meaningfully translated to the respective donor’s biofluids, several limitations must be acknowledged. First, the study focused exclusively on the LRRK2 G2019S mutation, and did not include other LRRK2 variants or familial PD-linked genes, nor did it systematically investigate sporadic PD beyond a single illustrative case. As such, the generalizability of our findings across the broader PD spectrum remains to be determined. In addition, our study focused exclusively on miRNAs; other classes of extracellular nucleic acids, such as cell-free DNA or long non-coding RNAs, were not analyzed but may also provide valuable complementary information.

Second, while we assessed the expression of LRRK2 and its kinase activity, mechanistic experiments targeting LRRK2 function, such as pharmacological inhibition, were not included. This decision was primarily due to substantial experimental constraints inherent to our model system. As outlined in the methods section, isolating EVs from iPSC-derived dopaminergic neurons requires large-scale cultures and extended collection of conditioned medium over a 9-day period. Sustained exposure to LRRK2 inhibitors like MLi-2 during this timeframe poses a challenge, as the compound is dissolved in DMSO, which accumulates and exerts cytotoxic or off-target effects with prolonged incubation. Nonetheless, we fully recognize the importance of assessing whether miRNA signatures are reversible upon LRRK2 inhibition, and this question should be addressed in future work using optimized protocols.

Third, while we explored potential downstream effects of let-7g-5p and miR-21-5p through GO enrichment and proteomic correlation, a more systematic mapping of the molecular pathways influenced by these miRNAs is still lacking.

Finally, although we performed careful validation of candidate miRNAs using independent EV preparations, small RNA libraries were generated at different time points across cell culture experiments, introducing a potential for batch effects. Nevertheless, validation experiments were restricted to RNA obtained within a consistent time window (days 14–23), aiming to minimize variability. In addition, normalization of RT-qPCR data remains a challenge in the miRNA field, as no universally accepted housekeeping control exists. We used miR-16 as a reference, based on its prior use in neurodegeneration studies, though its suitability may vary across sample types and disease contexts [71].

## Conclusions

This study highlights let-7g-5p and miR-21-5p as promising miRNA candidates in LRRK2-related PD. Using a patient-derived iPSC model and matched CSF samples, we demonstrate that cell-free miRNA alterations can reflect intracellular changes and may signal compensatory or pathogenic responses to the presence of PD pathology. Future studies may benefit from individualized miRNA profiling in patient-derived neurons and biofluids, which could help identify personalized molecular signatures and support the development of miRNA-based biomarker panels for Parkinson’s disease.

## Supplementary Information

Below is the link to the electronic supplementary material.

ESM1 (DOCX.1.18 MB)

## Data Availability

The datasets generated and analysed during the current study are available on NCBI, https://www.ncbi.nlm.nih.gov/geo/query/acc.cgi?acc=GSE243852. Competing Interests
