# Supplementary material for: Cellular and Extracellular MicroRNA Dysregulation in LRRK2-Linked Parkinson’s Disease
Source: Mol Neurobiol. 2025 Nov 27;63(1):189. doi: 10.1007/s12035-025-05379-2 (PMC12657546; doi:10.1007/s12035-025-05379-2)
Supplement: Supplementary file 1 — (DOCX.1.18 MB) [file 12035_2025_5379_MOESM1_ESM.docx]

**Supplemental Materials and Methods**

**Results**

**Immunocytochemistry of Neuronal and Dopaminergic Marker in the L2 Lines**

The number of MAP2-positive cells was observed to be 92.1% (SD: ±4.2%) in L2 GC and 90.8% (SD: ± 2.0%) in L2 Mut. On the other hand, the number of cells showing positive TH expression was 40.0% (SD: ±8.0%) in L2 GC and 40.2% (SD: ±5.5%) in L2 Mut (Supplemental Figure 1A and 1B). Ordinary Two-Way-ANOVA again showed no significant interaction between target and genotype (F(1, 8) = 0.050, p = 0.829). Simple main effects analysis showed that target (F(1, 8) = 271.9, p<0.0001) had a significant effect on the percentage of positively expressing cells but not genotype (F(1, 8) = 0.030, p = 0.867). Importantly, no significant difference in TH positive cells (mean difference: <0.01 p.p., q = 0.049, DF = 8, p>0.999) or MAP2 positive cells (mean difference: 0.01 p.p., q = 0.396, DF = 8, p = 0.992) was found between genotypes.

**mRNA Expression Levels of Neuronal and Dopaminergic Marker in the L2 Lines**

For LRRK2 mRNA levels, ordinary Two-Way-ANOVA in the L2 lines indicated a significant interaction between genotype and target (F3, 48) = 5.168, p = 0.004). Simple main effects analysis showed that both genotype and target had a statistically significant effect on the expression levels (target: F(3, 48) = 5.817, p = 0.002; genotype: F(1, 48) = 6.604, p = 0.013). After correction for multiple comparisons using Šidák’s test, the levels of LRRK2 mRNA were found to be significantly decreased in L2 Mut compared to L2 GC (t = 3.839, DF = 48, p = 0.0014). No significant difference between L2 Mut and L2 GC was found for any of the other gene expression levels (Supplemental Figure 1C).

**NTA Data for EVs derived from the L2 Lines**

The average size of particles in the L2 GC was 81.08 nm (SD: 7.54, n_Diff_ = 6) and 80.33 nm (SD: 9.01, n_Diff_ = 6) in L2 Mut.

**Quantification of pRab10 in the L2 lines**

In cell lysates, pRab10/Rab10 ratios did not differ significantly between genotypes (t(6) = 0.39, p = 0.71). Similarly, no significant difference was observed in EVs (t(10) = 0.82, p = 0.43) (Supplemental Figure 1H). When normalized to LRRK2 levels, pRab10/LRRK2 ratios showed a trend toward higher values in mutant lysates, though not statistically significant (t(6) = -2.00, p = 0.093). In contrast, a highly significant increase in normalized pRab10 levels was observed in mutant-derived EVs compared to controls (t(10) = -9.62, p < 0.001).

**Small-RNA Libraries Reveal a Subset of Differentially Expressed miRNAs in the L2 Lines**

In the L2 libraries, 2,608 miRNAs were identified, with 910 failing to meet the defined fold-change threshold. After applying multiple testing correction, 11 miRNAs were significantly upregulated and 14 were significantly downregulated in the mutant line compared to the isogenic control (Supplemental Figure 5, Supplemental Table 9). While a number of overlapping up- or downregulated miRNAs were noted between the L1 and L2 datasets prior to correction, no shared significantly dysregulated miRNAs remained after multiple testing correction (Supplemental Figure 6).

**Validation of Differentially Expressed miRNAs in the L2 Lines**

Of the seven miRNAs identified in the L1 libraries and subsequently tested in a new batch, two miRNAs were successfully validated in the L1 lines (log2 fold changes: miR-135a-5p = 0.59; miR-153-3p = 2.49) (Supplemental Figure 5C). Two miRNAs (miR-718 and miR-1234) were excluded due to unspecific amplification as indicated by the RT-qPCR melting curve. Supplemental Table 10 summarizes the RT-qPCR results using cell-free RNA obtained from the L1 lines. Next, we plotted the respective log2 fc values from the libraries against the log2 fc from the RT-qPCR experiments. Using Pearson’s correlation, we observed a moderate positive relationship between library fold-changes and RT-qPCR measurements in EV samples, although this did not reach statistical significance (r(8) = 0.54, p = 0.105, R² = 0.30) (Supplemental Figure 5D).

**Differentially Expressed Cell-Free RNAs Are Indicative of Changes in the L2 Cellular miRNAome**

In the L2 line, we similarly measured cellular miRNA levels by RT-qPCR and confirmed that the direction of dysregulation matched that seen in the EV preparations (Supplemental Figure 5E, Supplemental Table 11). To assess concordance between compartments in L2, we calculated Pearson’s correlation on the log₂ fold-change values. This analysis revealed a very strong positive association between cellular and cell-free RNA in L2 (r(8) = 0.94, p < 0.001, R² = 0.88), indicating highly concordant miRNA regulation across both matrices (Supplemental Figure 5E).

**Supplemental Figures**

**
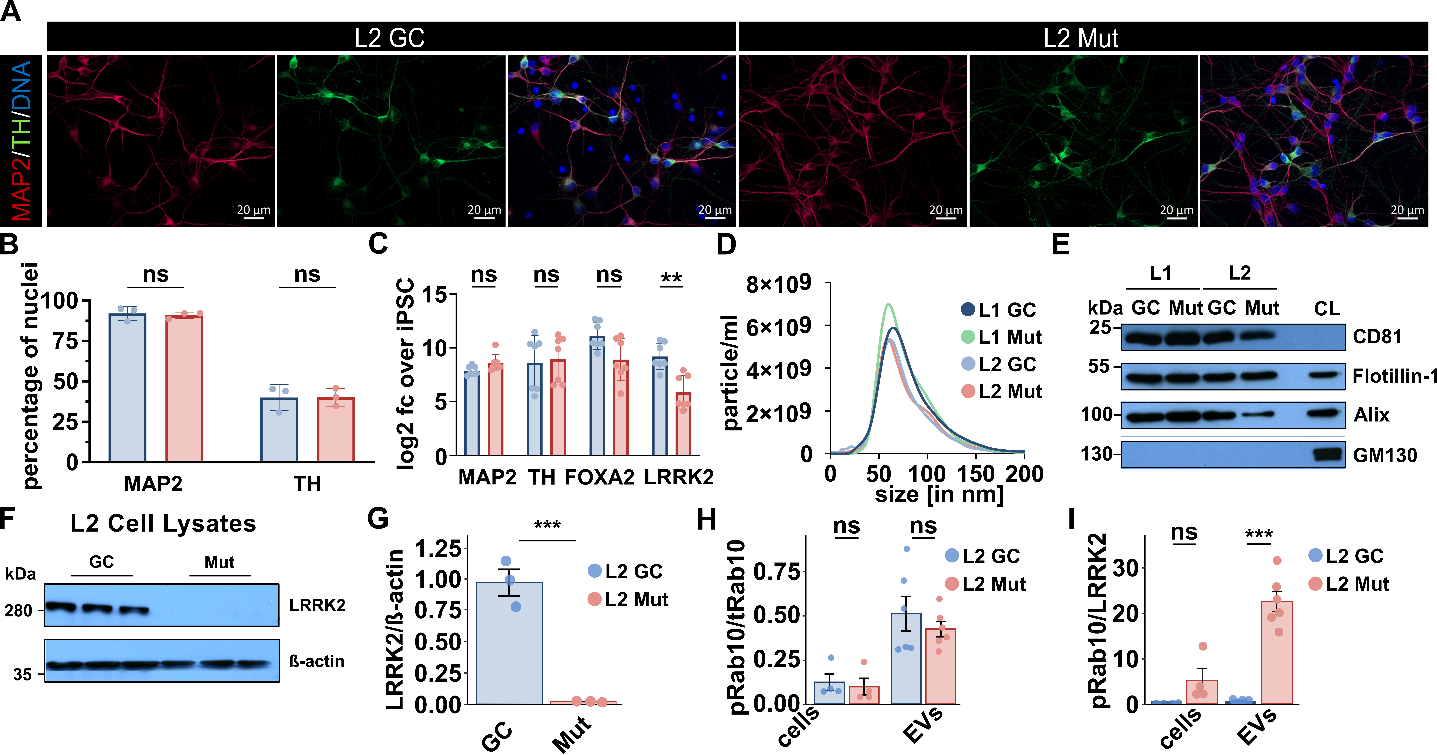
**

**Supplemental Figure 1│**Validation of neuronal identity in L2 lines and extracellular vesicles**.** **(A)** Representative images of immunofluorescence staining of hDaNs from the L2 lines on day 23 of the differentiation. Dopaminergic marker TH was stained together with neuronal marker MAP2 and DNA. **(B)** Percentages of MAP2+ and TH+ cells were calculated for both the L2 lines. 10 different positions were analyzed (n_Diff_ = 3). Error bars indicate standard deviation. **(C)** RT-qPCR was performed on cellular RNA isolated on day 23 from the L2 lines to quantify expression of MAP2, TH, FOXA2 and LRRK2 on mRNA level (n_Diff_ = 7). Results are shown as log2 fc of the expression in iPSC control samples. Error bars indicate standard deviation. **(D)** NTA measurements of EVs derived from all four lines. Size ranged between 30 to 200 nm. **(E)** Western blotting of CD81, Flotillin-1 and Alix revealed presence of vesicle markers in the EV samples. The Golgi apparatus associated protein GM130 was used as a negative control. Neuronal cell lysates (CL) were used as a positive control. **(F)** and **(G)** Western Blotting of LRRK2 in neuronal cell lysates from the L2 lines revealed a drastic downregulation of LRRK2 in the mutant line (p<0.001). **(H)** pRab10 to total Rab10 ratios in both cell lysates and EVs, with each data point representing an independent replicate. Kinase activity did not differ significantly between genotypes in either matrix. **(I)** Bar charts display pRab10 to total Rab10 ratios, normalized to the corresponding LRRK2 to β-Actin ratio. Following normalization, EVs from the mutant line exhibited significantly higher pRab10 levels compared to those from the isogenic gene-corrected control (p<0.001).

**
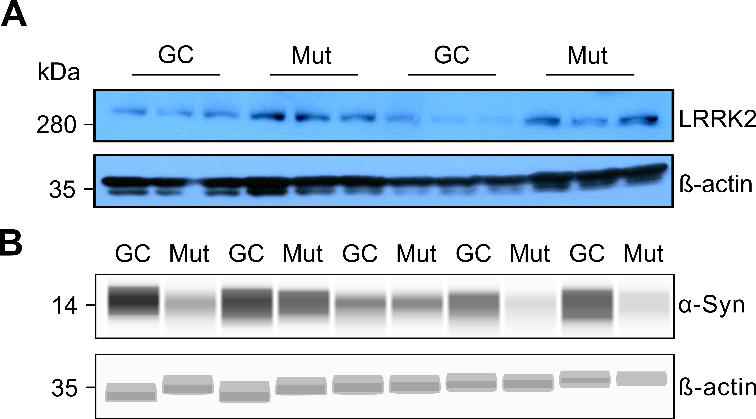
**

**Supplemental Figure 2│**Western Blots of LRRK2 and representative JESS images of α-synuclein using cell lysates from L1**.** **(A)** Cell lysates of two differentiations of hDaNs were generated. Each differentiation was blotted in technical replicates of three. For normalization, ß-actin was blotted as well. **(B)** For quantification of α-synuclein (α-Syn) levels, five differentiations were used and blotted using the JESS system. Again, ß-actin was used for normalization.


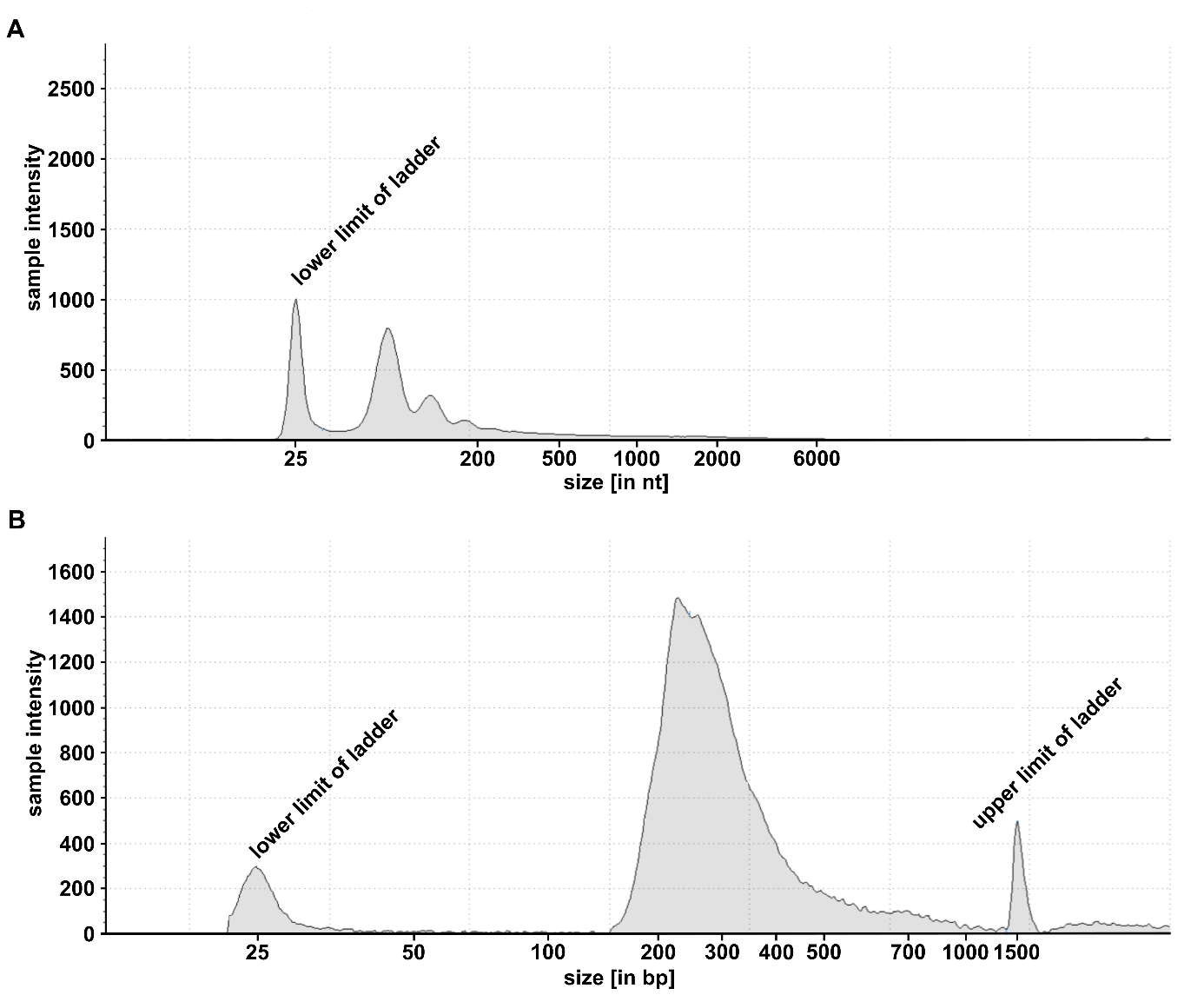


**Supplemental Figure 3│**Representative electropherograms of RNA input and library. **(A)** Electropherogram of cell-free RNA isolated from EVs derived from L2 Mut. Notably, no peak at 5000nt was observable, indicating the absence of rRNA. **(B)** Electropherogram of one of the L2 Mut libraries after the second bead purification. Sequencing of the libraries was done at a higher length to allow the inclusion and better mapping of potentially present mRNAs, which is not part of the present study.


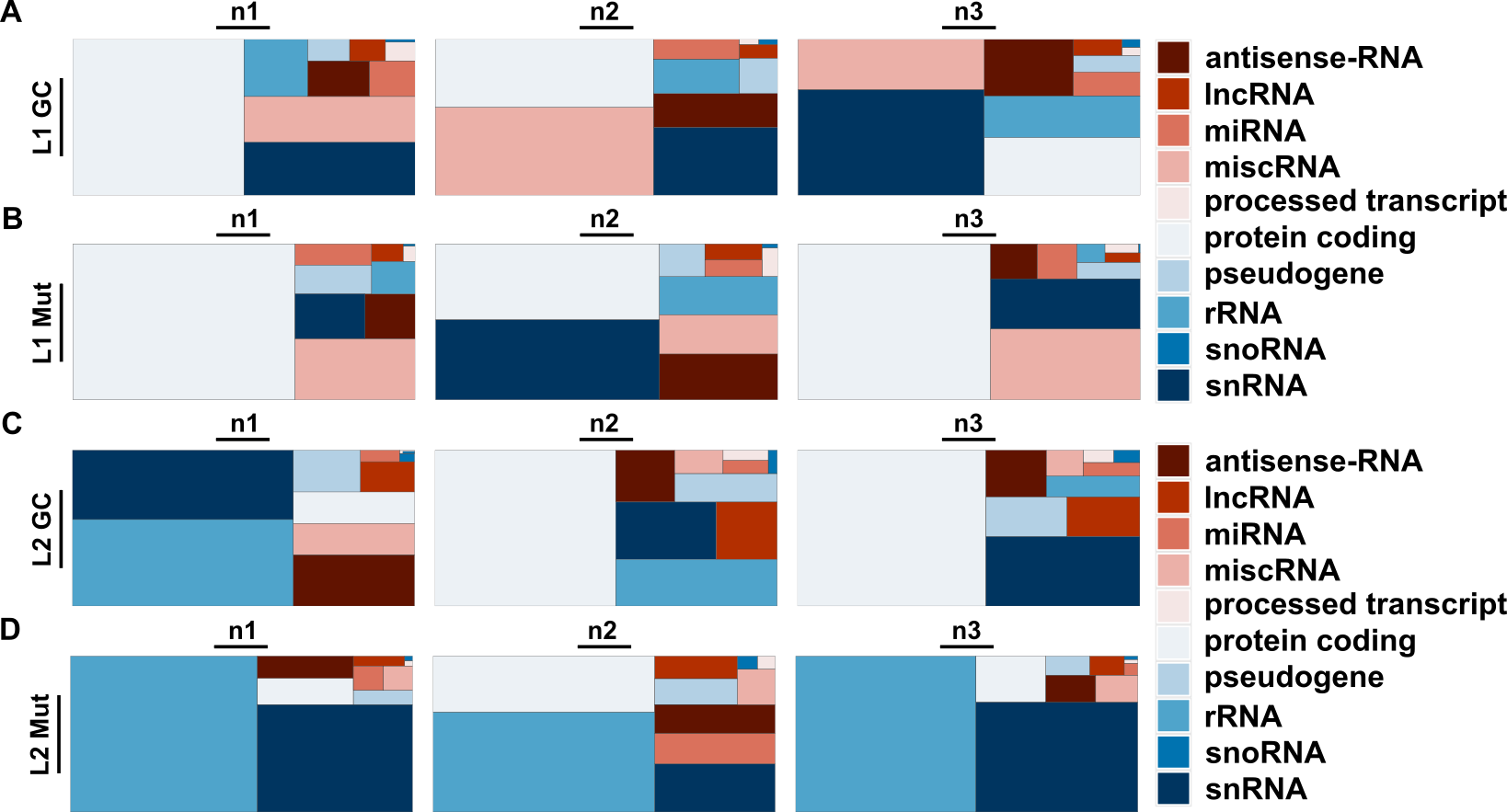


**Supplemental Figure 4│**Overview of biotype composition in the generated libraries. Size of the square represents the abundance of a given RNA biotype with higher abundance resulting in larger squares. Only the ten most abundant RNA biotypes are shown. The three boxes per row (n1-n3) represent the independent differentiations that were used to generate the libraries **(A)** Libraries generated from material from L1 GC, **(B)** L1 Mut, **(C)** L2 GC and **(D)** L2 Mut.


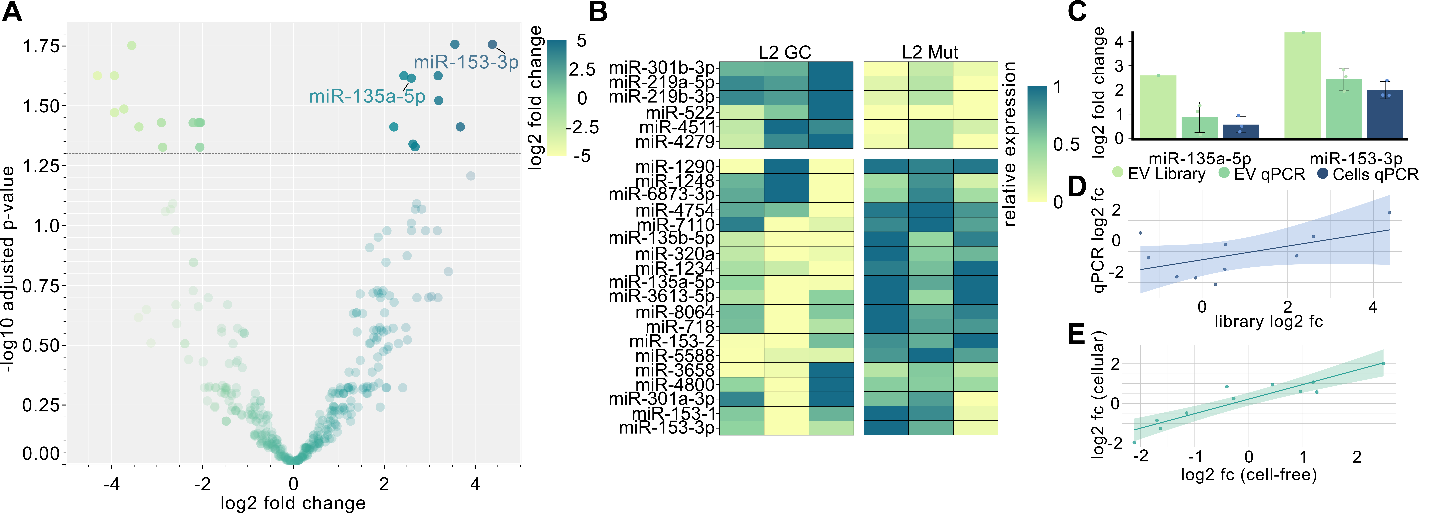


**Supplemental Figure 5│**Results of the small-RNA-libraries and validation RT-qPCR in the L2 lines**. (A)** A total of 2608 miRNAs were detected in the L2 libraries. 25 miRNAs passed the threshold and were considered differentially expressed. Log2 fc is expressed as change of L2 Mut over L2 GC. Horizontal line indicates the p-value threshold. For visualization purposes, the y-axis is capped at the 99th percentile to prevent extreme outliers from stretching the scale. **(B)** Heatmap visualizes the 25 miRNAs that were significantly differentially expressed. vst-transformed gene counts were normalized from 0 to 1 for each target, with 0 corresponding to the lowest observed expression and 1 to the highest. Colors reflect each sample’s relative expression level for a given miRNA. **(C)** RT-qPCR was conducted using both cell-free and cellular RNA. In L2, the dysregulation of miR-135a-5p and miR-153-3p was successfully validated. **(D)** Pearson’s correlation analysis revealed a strong directional correlation between miRNA log2 fold change levels measured in EV-derived RNA libraries and RT-qPCR (R² = 0.301, p = 0.0523). (**E**) Additionally, we conducted a correlation analysis of the log2 fold changes in miRNA expression between cellular and cell-free RNA. This analysis included data from miRNAs whose expression changes observed in the libraries could not be validated. We identified a significant correlation between the two compartments, indicating that alterations in cell-free miRNA expression reflect those in the cellular miRNAome.

**
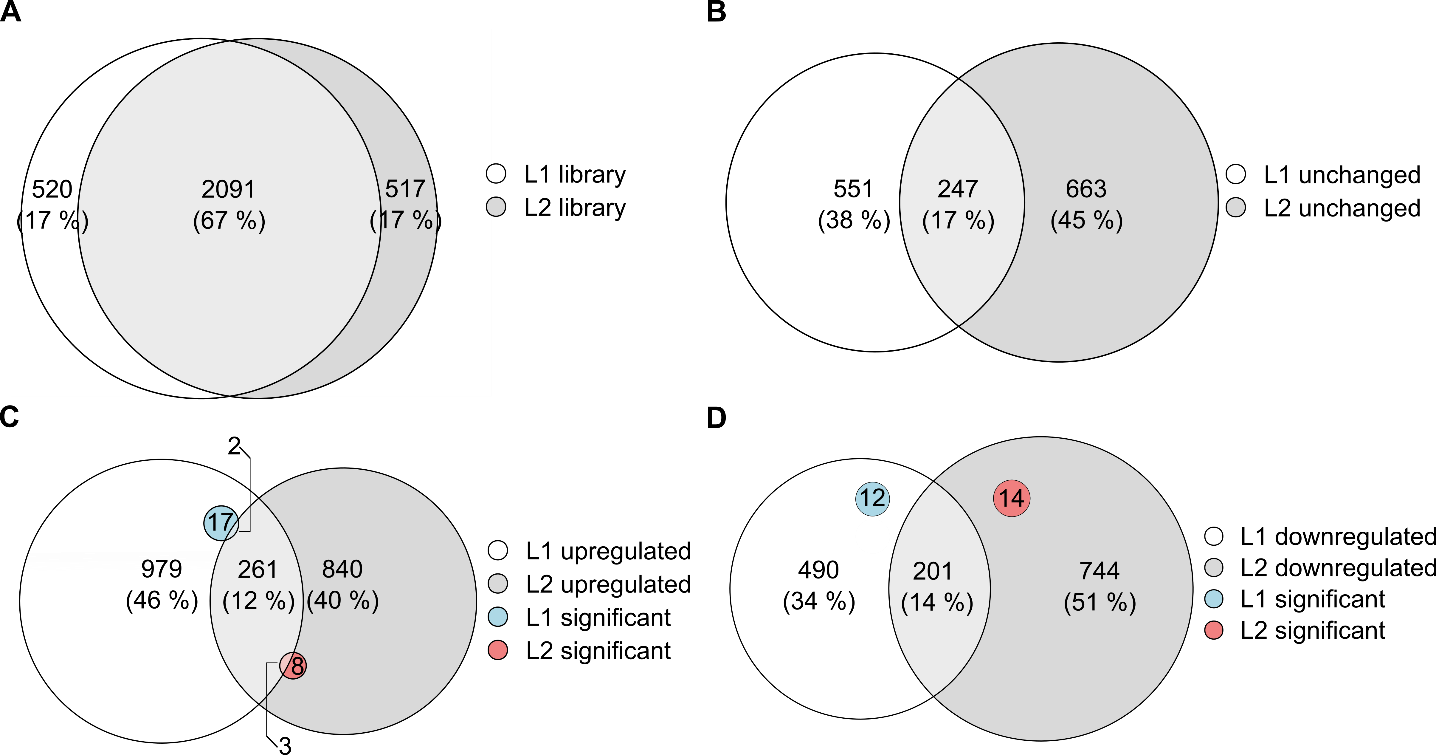
**

**Supplemental Figure 6│**Overview of overlaps between the L1 and L2 libraries. **(A)** A total of 2091 miRNAs were found to be present in both libraries **(B)** Of the miRNAs identified in the libraries, 798 (L1 Mut) and 910 (L2 Mut) did not show differential expression levels. Of these unchanged miRNAs, 247 were found in both patient lines. **(C)** Of the miRNAs found to be upregulated, 256 were shared by L1 Mut and L2 Mut. While there was no overlap after correction for multiple testing, 5 of the significantly upregulated miRNAs were among the 261 miRNAs that trended towards upregulation. **(D)** In L1 Mut, 703 miRNAs were downregulated compared to 959 in L2 Mut. Between them, 201 miRNAs were downregulated in both patient lines. After correction for multiple testing, no overlap between the lines was found.

**Supplemental Tables**

**Supplemental Table 1│** Sequences of primers used for the RT-qPCR based characterization of hDaNs.

| **Gene** | **Forward** | **Reverse** |
| --- | --- | --- |
| **GAPDH** | CGAGATCCCTCCAAAATCAAG | GCAGAGATGATGACCCTTTTG |
| **MAP2** | CCGTGTGGACCATGGGGCTG | GTCGTCGGGGTGATGCCACG |
| **TH** | TGTCTGAGGAGCCTGAGATTCG | GCTTGTCCTTGGCGTCACTG |
| **FOXA2** | CCATGCACTCGGCTTCCAG | TGTTGCTCACGGAGGAGT |
| **LRRK2** | TGTATCCCAATGCTGCCATC | ATTTCCTCTGGCAACTTCAGGT |

**Supplemental Table 2│** Overview of CNS terms used for filtering GO terms.

| Term | | | |
| --- | --- | --- | --- |
| alpha-synuclein | cerebrospinal fluid | myelin | oligodendrocyte |
| apoptosis | dopamine | nervous system | oxidative stress |
| astrocyte | dopaminergic neuron | neurodegeneration | plasticity |
| autophagy | gliosis | neurogenesis | protein aggregation |
| blood-brain barrier | grey matter | neuroinflammation | substantia nigra |
| brain | inflammation | neuron | synapse |
| brain development | microglia | neuroplasticity | tau protein |
| brain stem | midbrain | neuroprotection | white matter |
| central nervous system | mitochondria dysfunction | neurotransmitter |  |

**Supplemental Table 3│** Quality control data on the L1 libraries.

| **Library ID** | **L1 GC n_1_** | **L1 GC n_2_** | **L1 GC n_3_** | **L1 Mut n_1_** | **L1 Mut n_2_** | **L1 Mut n_3_** |
| --- | --- | --- | --- | --- | --- | --- |
| **% mapped (hairpin)** | 3.50 | 3.80 | 3.20 | 3.00 | 2.00 | 2.30 |
| **% mapped (mature)** | 0.90 | 1.70 | 2.40 | 1.60 | 2.80 | 4.10 |
| **% Duplicate Reads** | 38.70 | 43.90 | 46.50 | 44.20 | 56.00 | 60.40 |
| **% GC** | 62.00 | 60.00 | 61.00 | 58.00 | 56.00 | 53.00 |

**Supplemental Table 4│** Quality control data on the L2 libraries.

| **Library ID** | **L2 GC n_1_** | **L2 GC n_2_** | **L2 GC n_3_** | **L2 Mut n_1_** | **L2 Mut n_2_** | **L2 Mut n_3_** |
| --- | --- | --- | --- | --- | --- | --- |
| **% Mapped (hairpin)** | 2.10 | 5.10 | 7.30 | 1.50 | 5.20 | 1.50 |
| **% Mapped (mature)** | 2.30 | 0.80 | 1.00 | 1.00 | 0.70 | 2.10 |
| **% Duplicate Reads** | 60.10 | 46.80 | 70.10 | 61.80 | 49.40 | 62.80 |
| **% GC** | 54.00 | 51.00 | 53.00 | 50.00 | 56.00 | 48.00 |

**Supplemental Table 5│** Overview of library biotype composition in libraries from the L1 lines. Numbers are given as percentages.

| **biotype** | **L1 GC n_1_** | **L1 GC n_2_** | **L1 GC n_3_** | **L1 Mut n_1_** | **L1 Mut n_2_** | **L1 Mut n_3_** |
| --- | --- | --- | --- | --- | --- | --- |
| **antisense-RNA** | 4.20 | 10.12 | 3.06 | 4.10 | 7.86 | 9.40 |
| **lncRNA** | 1.05 | 1.73 | 0.62 | 1.45 | 0.99 | 1.47 |
| **miRNA** | 3.03 | 1.77 | 2.59 | 3.03 | 3.21 | 2.94 |
| **misc_RNA** | 13.73 | 8.69 | 19.86 | 14.53 | 35.77 | 17.54 |
| **Mt_rRNA** | 0.01 | 0.18 | 0.01 | 0.07 | 0.07 | 0.12 |
| **Mt_tRNA** | 0.02 | 0.03 | 0.01 | 0.01 | 0.01 | 0.01 |
| **processed transcript** | 0.35 | 0.85 | 0.57 | 1.04 | 0.19 | 0.28 |
| **protein coding** | 64.87 | 31.74 | 56.13 | 49.90 | 27.74 | 16.81 |
| **pseudogene** | 4.14 | 2.78 | 1.96 | 1.70 | 2.54 | 2.07 |
| **rRNA** | 2.64 | 8.38 | 0.96 | 6.81 | 5.45 | 12.15 |
| **sense_intronic** | 0.03 | 0.20 | 0.16 | 0.12 | 0.22 | 0.30 |
| **snoRNA** | 0.05 | 0.12 | 0.03 | 0.16 | 0.18 | 0.27 |
| **snRNA** | 5.87 | 33.42 | 14.05 | 17.06 | 15.75 | 36.66 |

**Supplemental Table 6│** Overview of library biotype composition in libraries from the L2 lines. Numbers are given as percentages.

| **biotype** | **L2 GC n_1_** | **L2 GC n_2_** | **L2 GC n_3_** | **L2 Mut n_1_** | **L2 Mut n_2_** | **L2 Mut n_3_** |
| --- | --- | --- | --- | --- | --- | --- |
| **antisense-RNA** | 11.59 | 5.68 | 5.15 | 4.02 | 6.46 | 2.52 |
| **lncRNA** | 3.06 | 6.51 | 5.30 | 0.97 | 3.49 | 1.25 |
| **miRNA** | 0.85 | 1.09 | 1.40 | 1.37 | 6.79 | 0.30 |
| **misc_RNA** | 7.13 | 2.12 | 1.75 | 1.33 | 2.48 | 2.14 |
| **Mt_rRNA** | 0.13 | 0.39 | 1.74 | 0.05 | 0.67 | 0.06 |
| **Mt_tRNA** | 0.01 | 0.03 | 0.01 | <0.01 | 0.01 | 0.01 |
| **processed transcript** | 0.10 | 0.88 | 0.71 | 0.08 | 0.45 | 0.10 |
| **protein coding** | 7.12 | 52.22 | 53.77 | 4.74 | 23.11 | 6.08 |
| **pseudogene** | 5.26 | 5.41 | 5.90 | 1.60 | 3.97 | 1.59 |
| **rRNA** | 35.68 | 13.95 | 3.56 | 54.52 | 40.95 | 52.62 |
| **sense_intronic** | 0.16 | 0.66 | 0.57 | 0.05 | 0.39 | 0.07 |
| **snoRNA** | 0.23 | 0.39 | 0.60 | 0.07 | 0.49 | 0.09 |
| **snRNA** | 28.68 | 10.67 | 19.55 | 31.20 | 10.74 | 33.17 |

**Supplemental Table 7│**Significantly dysregulated miRNAs in the L1 Libraries.

| **miRNA** | **log2fc** | **adjusted p-value** |
| --- | --- | --- |
| **miR-424-3p** | 4.74 | 0.0465 |
| **let-7g-5p** | 4.55 | <0.0001 |
| **let-7i-5p** | 4.35 | 0.0034 |
| **miR-98-3p** | 4.29 | 0.0429 |
| **let-7f-2-3p** | 4.02 | 0.0269 |
| **let-7d-3p** | 3.74 | 0.0269 |
| **let-7a-5p** | 3.54 | 0.0327 |
| **let-7g** | 3.48 | 0.0133 |
| **let-7e-5p** | 3.46 | 0.0133 |
| **miR-4330** | 2.88 | 0.0043 |
| **miR-21-5p** | 2.78 | 0.0002 |
| **miR-21** | 2.67 | 0.0327 |
| **miR-29a-3p** | 2.62 | 0.0269 |
| **miR-128-3p** | 2.28 | 0.0192 |
| **miR-27a-3p** | 2.20 | 0.0244 |
| **miR-24-1** | 2.08 | 0.0115 |
| **miR-3074** | 2.08 | 0.0115 |
| **miR-24-2** | 2.07 | 0.0120 |
| **miR-24-3p** | 1.83 | 0.0442 |
| **miR-12121** | -1.74 | 0.0327 |
| **miR-4449** | -1.81 | 0.0269 |
| **miR-1268a** | -1.92 | 0.0442 |
| **miR-4466** | -2.00 | 0.0463 |
| **miR-4787-5p** | -2.35 | 0.0036 |
| **miR-4488** | -2.90 | 0.0036 |
| **miR-4787** | -2.94 | 0.0002 |
| **miR-6724-1** | -3.49 | 0.0011 |
| **miR-6724-2** | -3.49 | 0.0011 |
| **miR-6724-3** | -3.49 | 0.0011 |
| **miR-6724-4** | -3.49 | 0.0011 |
| **miR-10398** | -3.57 | 0.0015 |

**Supplemental Table 8│**Significantly dysregulated miRNAs in the L2 Library.

| **miRNA** | **log2fc** | **adjusted p-value** |
| --- | --- | --- |
| **miR-135b-5p** | 5.21 | 0.0165 |
| **miR-153-3p** | 4.38 | 0.0015 |
| **miR-3613-5p** | 3.68 | 0.0387 |
| **miR-1234** | 3.56 | 0.0018 |
| **miR-718** | 3.20 | 0.0300 |
| **miR-153-1** | 3.19 | 0.0237 |
| **miR-153-2** | 2.68 | 0.0469 |
| **miR-8064** | 2.63 | 0.0457 |
| **miR-135a-5p** | 2.60 | 0.0242 |
| **miR-4754** | 2.43 | 0.0237 |
| **miR-320a** | 2.21 | 0.0387 |
| **miR-301b-3p** | -2.04 | 0.0372 |
| **miR-1290** | -2.05 | 0.0471 |
| **miR-219b-3p** | -2.06 | 0.0372 |
| **miR-219a-5p** | -2.08 | 0.0372 |
| **miR-301a-3p** | -2.20 | 0.0372 |
| **miR-4800** | -2.87 | 0.0471 |
| **miRr-7110** | -2.90 | 0.0372 |
| **miR-6873-3p** | -3.40 | 0.0387 |
| **miR-5588** | -3.55 | 0.0177 |
| **miR-4279** | -3.73 | 0.0326 |
| **miR-1248** | -3.93 | 0.0338 |
| **miR-3658** | -3.94 | 0.0237 |
| **miR-4511** | -4.31 | 0.0237 |
| **miR-522** | -6.83 | 0.0165 |

**Supplemental Table 9│List of miRNAs for validation via RT-qPCR.** From each library, seven miRNAs were selected for quantification in cell-free and cellular RNA. Log2 fold change and adjusted p-value from the library are depicted. miRNAs denoted in bold were found to be differentially expressed in the respective library.

| **miRNA** | **log 2 fc L1** | **adj. p-value L1** | **log 2 fc L2** | **adj. p-value L2** |
| --- | --- | --- | --- | --- |
| **let-7g-5p** | **4.55** | <0.0001 | 0.53 | 0.76 |
| **miR-1234** | -1.04 | 0.39 | **3.56** | 0.001 |
| **miR-128-3p** | **2.28** | 0.019 | 0.55 | 0.72 |
| **miR-135a-5p** | 0.51 | 0.66 | **2.6** | 0.024 |
| **miR-153-3p** | 0.54 | 0.6 | **4.38** | 0.0015 |
| **miR-21-5p** | **2.78** | 0.0002 | 0.31 | 0.86 |
| **miR-219a-5p** | 0.18 | 0.91 | **-2.08** | 0.037 |
| **miR-24-3p** | **1.83** | 0.044 | -0.59 | 0.73 |
| **miR-27a-3p** | **2.2** | 0.024 | -0.15 | 0.95 |
| **miR-29a-3p** | **2.62** | 0.027 | 0.48 | 0.83 |
| **miR-301a-3p** | 1.33 | 0.52 | **-2.2** | 0.037 |
| **miR-320a** | -1.37 | 0.3 | **2.21** | 0.038 |
| **miR-424-3p** | **4.74** | 0.046 | -0.07 | NA |
| **miR-718** | -1.14 | 0.38 | **3.2** | 0.03 |

**Supplemental Table 10│**Overview of the validation RT-qPCRs in cell-free RNA. Displayed miRNAs were identified in the L2 libraries. Targets where dysregulation crossed the fc threshold of either ≥1.5 or ≤0.5 over the gene corrected control are highlighted in bold. A * highlights those miRNAs, where dysregulation found in the library was confirmed.

| target | fc (L2) |
| --- | --- |
| miR-135a-5p* | **1.94 (SD ± 0.73)** |
| miR-219a-5p | **2.28 (SD ± 0.57)** |
| miR-301a-3p | 0.75 (SD ±0.10) |
| miR-320a | 0.82 (SD ±0.04) |
| miR-153a-3p* | **5.62 (SD** ±**1.67)** |

**Supplemental Table 11│**Overview of the RT-qPCRs results using cellular RNA. Displayed miRNAs were identified in the L2 libraries. Targets where dysregulation crossed the threshold of either ≥1.5 or ≤0.5 over the gene corrected control are highlighted in bold.

| target | fc (L2) |
| --- | --- |
| miR-135a-5p | **1.51 (SD ±0.37)** |
| miR-219a-5p | **2.10 (SD ±0.66)** |
| miR-301a-3p | **1.80 (SD ±0.37)** |
| miR-320a | 1.19 (SD ±0.36) |
| miR-153a-3p | **4.05 (SD ±1.07)** |

**Supplemental Table 12│List of proteins targeted by let-7g-5p an miR-21-5p, respectively.**

| let-7g-5p | miR-21-5p | | | |
| --- | --- | --- | --- | --- |
| AGO1 | ACAT1 | FOXO3 | PELI1 | STRN |
| AKT2 | AKT2 | GAS5 | PFKFB2 | TCF21 |
| BCL2L1 | AMH | GDF5 | PIAS3 | TGFBI |
| BMI1 | ANKRD46 | GLS | PLAT | TGFBR2 |
| CASP3 | ANP32A | GLS2 | PLOD3 | TGFBR3 |
| CDKN2A | APAF1 | HIPK3 | PPARA | TGIF1 |
| COL1A2 | BASP1 | HNRNPK | PPIF | TIAM1 |
| FN1 | BCL2 | HPGD | PTEN | TIMP3 |
| GAB2 | BCL6 | ICAM1 | PTX3 | TLR2 |
| HMGA2 | BMPR2 | IGF1R | RASA1 | TM9SF3 |
| HOXB1 | BTG2 | IL12A | RASGRP1 | TNFAIP3 |
| IGF2BP1 | CASC2 | IL1B | RECK | TNFRSF10B |
| IL13 | CBX4 | INTU | REST | TOPORS |
| KRAS | CCL20 | IRAK1 | RFFL | TOR1AIP2 |
| MMP16 | CCR1 | ISCU | RHO | TP53BP2 |
| MYC | CCR7 | JAG1 | RHOB | TP63 |
| SMAD2 | CDC25A | JMY | RMND5A | TPM1 |
| TBC1D9 | CDIP1 | KIF27 | RPS7 | VEGFA |
| TGFBR1 | CDK2AP1 | Lats1 | RTN4 | VHL |
| THBS1 | CDK6 | LATS2 | SASH1 | WWC2 |
| TNFRSF10B | CLU | LRRFIP1 | SATB1 | WWP1 |
|  | COL4A1 | MAP2K3 | SECISBP2L | YOD1 |
|  | CXCL10 | MARCKS | SERPINB5 |  |
|  | DAXX | MEF2C | SERPINI1 |  |
|  | DDAH1 | MKNK2 | SETD2 |  |
|  | DERL1 | MMP2 | SIRT2 |  |
|  | DNM1L | MMP9 | SLC16A10 |  |
|  | DOCK4 | MSH2 | SMAD7 |  |
|  | DOCK5 | MSH6 | SMARCA4 |  |
|  | DOCK7 | MTAP | SMN1 |  |
|  | DUSP10 | MYC | SOD3 |  |
|  | E2F1 | MYD88 | SOX17 |  |
|  | E2F2 | NCAPG | SOX2 |  |
|  | EGFR | NCOA3 | SOX5 |  |
|  | EIF4A2 | NFIA | SOX6 |  |
|  | ELAVL4 | NFIB | SP1 |  |
|  | ENO4 | NTF3 | SPRY1 |  |
|  | ERBB2 | PACRG | SPRY2 |  |
|  | FASLG | PCBP1 | STAT3 |  |
|  | FMOD | PDCD4 | STK36 |  |

**Supplemental Table 13│**Overview of CNS-related GO terms significantly associated to proteins targeted by let-7g-5p

| ID | Description | pvalue | p.adjust | Gene Count |
| --- | --- | --- | --- | --- |
| GO:0097194 | execution phase of apoptosis | 1,3417E-04 | 4,1874E-03 | 3 |
| GO:0051402 | neuron apoptotic process | 2,6471E-04 | 6,3435E-03 | 4 |
| GO:0048483 | autonomic nervous system development | 1,0612E-03 | 1,2716E-02 | 2 |
| GO:0043523 | regulation of neuron apoptotic process | 2,2794E-03 | 1,8539E-02 | 3 |
| GO:0030900 | forebrain development | 9,0243E-03 | 4,5358E-02 | 3 |

**Supplemental Table 14│**Overview of CNS-related GO terms significantly associated to proteins targeted by miR-21-5p

| ID | Description | pvalue | p.adjust | Gene Count |
| --- | --- | --- | --- | --- |
| GO:0051402 | neuron apoptotic process | 7,00E+05 | 1,00E+08 | 15 |
| GO:0043523 | regulation of neuron apoptotic process | 2,55E+08 | 8,31E+09 | 11 |
| GO:0050767 | regulation of neurogenesis | 4,54E+08 | 0.000129003262007311 | 13 |
| GO:0030900 | forebrain development | 1,10E+09 | 0.000249901074323499 | 13 |
| GO:0051961 | negative regulation of nervous system development | 1,15E+09 | 0.000257644555178578 | 8 |
| GO:0045664 | regulation of neuron differentiation | 1,24E+09 | 0.000270074269573585 | 9 |
| GO:0097150 | neuronal stem cell population maintenance | 3,00E+09 | 0.000514798268803925 | 4 |
| GO:0051960 | regulation of nervous system development | 3,38E+09 | 0.000563527607013743 | 13 |
| GO:0043524 | negative regulation of neuron apoptotic process | 3,46E+09 | 0.00056926048204602 | 8 |
| GO:0050768 | negative regulation of neurogenesis | 6,75E+09 | 0.000932675050790246 | 7 |
| GO:0048709 | oligodendrocyte differentiation | 0.000152436990211374 | 0.00172890561049862 | 6 |
| GO:0006979 | response to oxidative stress | 0.000185338161429402 | 0.00204385221711685 | 11 |
| GO:0043217 | myelin maintenance | 0.000283031975628903 | 0.00269068063833949 | 3 |
| GO:0034599 | cellular response to oxidative stress | 0.000505505301754995 | 0.00431364524164262 | 8 |
| GO:0097194 | execution phase of apoptosis | 0.000678830451525533 | 0.00542775236871102 | 5 |
| GO:0022010 | central nervous system myelination | 0.000967153033271719 | 0.00700257872978958 | 3 |
| GO:0032291 | axon ensheathment in central nervous system | 0.000967153033271719 | 0.00700257872978958 | 3 |
| GO:0042552 | myelination | 0.00105193749615091 | 0.00757057025342344 | 6 |
| GO:0007272 | ensheathment of neurons | 0.00112247029712969 | 0.00793478016748087 | 6 |
| GO:0045665 | negative regulation of neuron differentiation | 0.00197298132285858 | 0.0123059804137593 | 4 |
| GO:1900117 | regulation of execution phase of apoptosis | 0.0024365663270484 | 0.014222563055605 | 3 |
| GO:0007422 | peripheral nervous system development | 0.0037318902343632 | 0.0194405444766827 | 4 |
| GO:0071679 | commissural neuron axon guidance | 0.00388189810856825 | 0.0198753183158694 | 2 |
| GO:0014002 | astrocyte development | 0.00400233865155948 | 0.0202890830653312 | 3 |
| GO:0048708 | astrocyte differentiation | 0.00421305682188613 | 0.0212072972607302 | 4 |
| GO:0014004 | microglia differentiation | 0.0051761587502635 | 0.0241239960480421 | 2 |
| GO:1900119 | positive regulation of execution phase of apoptosis | 0.0051761587502635 | 0.0241239960480421 | 2 |
| GO:0010507 | negative regulation of autophagy | 0.00529764952005298 | 0.024467494690554 | 4 |
| GO:0045666 | positive regulation of neuron differentiation | 0.00529764952005298 | 0.024467494690554 | 4 |
| GO:0014003 | oligodendrocyte development | 0.0057386966954126 | 0.0257737956846601 | 3 |
| GO:0050769 | positive regulation of neurogenesis | 0.0094333852410354 | 0.0361209964784945 | 6 |
| GO:0031102 | neuron projection regeneration | 0.00994552176868609 | 0.0375246765393769 | 3 |
| GO:1902284 | neuron projection extension involved in neuron projection guidance | 0.010061722699314 | 0.0375246765393769 | 2 |
| GO:0036480 | neuron intrinsic apoptotic signaling pathway in response to oxidative stress | 0.0120077422106146 | 0.042736591939268 | 2 |
| GO:0008631 | intrinsic apoptotic signaling pathway in response to oxidative stress | 0.0123182603948167 | 0.043539097884638 | 3 |
| GO:0043525 | positive regulation of neuron apoptotic process | 0.0144303473518187 | 0.0481997809029994 | 3 |
| GO:0048143 | astrocyte activation | 0.015207745887903 | 0.0495045969684325 | 2 |

**Supplemental Table 15│**Overview of Clinical Data. Of note, all individuals were female.

|  |  | | **L1** | | **L2** | | **L3** | | **L4** | | **L5** | |
| --- | --- | --- | --- | --- | --- | --- | --- | --- | --- | --- | --- | --- |
|  | **Patient** | **sPD** | | **Control** | **Patient** | **Control** | **Patient** | **Control** | **Patient** | **Control** | **Patient** | **Control** |
| **Age at Study** | 51 | 51 | | 57 | 80 | 84 | 66 | 67 | 79 | 76 | 63 | 59 |
| **Age at Onset** | 40 | 44 | | - | 70 | - | 53 | - | 70 | - | 59 | - |
| **Disease Duration** | 11 | 6 | | - | 10 | - | 13 | - | 9 | - | 4 | - |

**Supplemental Table 16│**Overview of fold-change values of let-7g-5p in CSF derived from patients with the LRRK2 G2019S mutation.

| Patient | FC | SD |
| --- | --- | --- |
| L1 | 1.92 | ±0.707 |
| L2 | 2.30 | ±1.97 |
| L3 | 0.419 | ±0.235 |
| L4 | 0.235 | ±0.0765 |
| L5 | 0.613 | ±0.651 |

**Supplemental Table 17│** Overview of fold-change values of miR-21-5pin CSF derived from patients with the LRRK2 G2019S mutation.

| Patient | FC | SD |
| --- | --- | --- |
| L1 | 60.5 | ±5.31 |
| L2 | 15.5 | ±1.35 |
| L3 | 0.541 | ±0.108 |
| L4 | 0.127 | ±0.0520 |
| 5 | 0.606 | ±0.114 |
